# The effect and comparison of training in ethical decision-making through lectures and group discussions on moral reasoning, moral distress and moral sensitivity in nurses: a clinical randomized controlled trial

**DOI:** 10.1186/s12910-023-00938-5

**Published:** 2023-08-04

**Authors:** Morteza Khaghanizadeh, Aliakbar Koohi, Abbas Ebadi, Amir Vahedian-Azimi

**Affiliations:** 1 Behavioral Sciences Research Center, Life Style Institute, Nursing Faculty, Baqiyatallah University of Medical Sciences, Tehran, Iran; 2 Trauma Research Center, Nursing Faculty, Baqiyatallah University of Medical Sciences, Tehran, Iran; 3 Trauma Research Center, Nursing Faculty, Baqiyatallah University of Medical Sciences, Sheykh bahayi Street, Vanak Square Tehran, Tehran, P.O. Box 19575-174, Iran

**Keywords:** Ethical decision-making, Moral reasoning, Moral distress, Moral sensitivity, Nurses

## Abstract

**Background:**

Ethical decision‑making and behavior of nurses are major factors that can affect the quality of nursing care. Moral development of nurses to making better ethical decision-making is an essential element for managing the care process. The main aim of this study was to examine and comparison the effect of training in ethical decision-making through lectures and group discussions on nurses’ moral reasoning, moral distress and moral sensitivity.

**Methods:**

In this randomized clinical trial study with a pre- and post-test design, 66 nurses with moral reasoning scores lower than the average of the community were randomly assigned into three equal groups (n = 22) including two experimental groups and one control group. Ethical decision-making training to experimental groups was provided through the lectures and group discussions. While, the control group did not receive any training. Data were collected using sociodemographic questionnaire, the nursing dilemma test (NDT), the moral distress scale (MDS) and the moral sensitivity questionnaire (MSQ). Unadjusted and adjusted binary logistic regression analysis was reported using the odds ratio (OR) and 95% confidence intervals.

**Results:**

Adjusted regression analysis showed that the probability of increasing the nursing principle thinking (NPT) score through discussion training was significantly higher than lecture (OR: 13.078, 95% CI: 3.238–15.954, P = 0.008), as well as lecture (OR: 14.329, 95% CI: 16.171–2.005, P < 0.001) and discussion groups compared to the control group (OR: 18.01, 95% CI: 22.15–5.834, P < 0.001). The possibility of increasing moral sensitivity score through discussion training was significantly higher than lecture (OR: 10.874, 95%CI: 6.043–12.886, P = 0.005) and control group (OR: 13.077, 95%CI: 8.454–16.774, P = 0.002). Moreover, the moral distress score was significantly reduced only in the trained group compared to the control, and no significant difference was observed between the experimental groups; lecture group vs. control group (OR: 0.105, 95% CI: 0.015–0.717, P = 0.021) and discussion group vs. control group (OR: 0.089, 95% CI: 0.015–0.547, P = 0.009).

**Conclusions:**

The results of this study indicate that ethical decision-making training is effective on empowerment of ethical reasoning. Whereas the group discussion was also effective on increasing the ethical sensitivity, it is recommended the training plan provided in this study to be held as workshop for all nurses in health and treatment centers and placed in curricular plan of nursing students.

**Registration:**

This randomized clinical trial was registered in Iranian Registry of Clinical Trials under code (IRCT2015122116163N5) in 02/07/2016.

**Supplementary Information:**

The online version contains supplementary material available at 10.1186/s12910-023-00938-5.

## Background

Patient care is an important concept and in fact the art of the nursing profession and requires the personal, social, moral, and spiritual ability of the nurse to be able to provide desirable and ethical care [1, 2]. In the patient care process, commitment and observance of ethical takes precedence over caring [3]. Ethical dimension of nursing care is an essential component in nursing practice [4, 5]. Advances in science and technology have made patient care more sophisticated, so nurses face difficult situations in patient care that require appropriate ethical decisions [6, 7]. The ethical decision-making process for nurses is a challenging process that can be influenced by several factors, including sociodemographic characteristics, ability of moral reasoning, moral sensitivity, and moral distress [8–10]. Ethical decision-making is a logical process which involves making the best moral decisions through systematic reasoning in a situation that brings about conflicting choices [11, 12].

One of the components of the moral decision-making process is moral reasoning, which refers to the ability of an individual to make judgments and make correct and rational decisions in dealing with everyday ethical dilemmas [13, 14]. Familiarity of nurses with ethical dilemmas during their professional activities makes them think about the consequences when making decisions, respect people and perform their professional duties by considering principles such as honesty, confidentiality and fairness [15, 16]. Moral sensitivity in nurses makes them use ethics better and more effectively in caring for clients [8]. Moral sensitivity is a combination of one’s knowledge of dimensions ethics and includes responsibility, giving importance to the issues, tolerance, and tranquility [17, 18]. Moral distress is a common phenomenon in nursing practice that can cause conflict in dealing with patients and providing quality care. It can disrupt the process of achieving the goals of the care system, such as making the correct moral decisions in the face of daily dilemmas, and thus have an adverse effect on the pattern of community health [19, 20]. Therefore, understanding the effects of sociodemographic factors, abilities of moral reasoning, moral sensitivities and moral distress on nurses’ ethical behavior provides valuable data for policy makers, based on which they can design programs to improve nurses’ ethical performance. Since there is no complete and proven information about the impact of these factors on nurses’ moral performance, in this study, the relationship between moral reasoning, moral sensitivity and moral distress levels of the nurses and their sociodemographic characteristics was investigated.

In addition, moral development of nurses is an essential element for managing the care process as qualified and efficient [21]. However, it seems that making ethical decision in the presence of daily moral dilemmas is very difficult [15, 22]. Thus, strategies are needed to improve nurses’ ethical decision‑making to minimize the likelihood of these problems [23]. It seems that one of the ways to improve the level of ethical decision-making in nurses is training. So that it can increase the ability of moral reasoning and moral sensitivity of nurses and reduce their moral distress. In this regard, some previous studies have reported that ethical decision-making programs through purely theoretical training such as lecture method are not entirely satisfactory [4, 24]. Accordingly, it seems that the active learning strategies are needed to improve the outcomes of nursing ethics education. Evidence showed that the strategies such as case‑based learning (CBL) [15], simulation [25], exposure to challenging situations [26], and multimedia education can be more effective [27]. One of the active learning strategies is group discussions (GD) technique is often used as a qualitative approach to gain in-depth understanding of issues [28]. According to our literature search, the impact of nursing ethics education through GD on ethical reasoning, distress, and sensitivity is still unclear as to whether GD can be more effective in improving these abilities than lecture method or not. In this study, the effect of training in ethical decision-making through lectures and group discussions on nurses’ moral reasoning, moral distress and moral sensitivity was examined and compared.

## Methods

### Trial design

This randomized clinical trial study with a pre- and post-test design was conducted to examine and comparison the effect of training in ethical decision-making through lectures and group discussions on nurses’ moral reasoning, moral distress and moral sensitivity. The study protocol was reviewed and approved by the Ethics Committee of Baqiyatallah University of Medical Sciences, Tehran, Iran, under code IR.BMSU.REC.1394.145, in accordance with the Declaration of Helsinki of the World Medical Association [29]. This randomized clinical trial study was registered in Iranian Registry of Clinical Trials under code (IRCT2015122116163N5) in 02/07/2016. Written informed consent was obtained from all participants. This study was performed and reported in accordance with the recommendations of the Consolidated Standards of Reporting Trials (CONSORT) statement [30].

### Setting and participants

All nurses of Baqiyatallah Hospital in Tehran, Iran, were eligible to participate in the study if they met all the inclusion criteria. The study inclusion criteria for nurses included having bachelor’s or higher degrees in nursing, having at least 1 year of work experience in direct participation in patient care. Based on inclusion criteria, 270 nurses were selected by census method in 2015, and the levels of moral reasoning, moral distress, and moral sensitivity of these nurses were determined by questionnaires. Out of 270 questionnaires which distributed among participants, 25 questionnaires were excluded due to incompleteness. Therefore, the final sample size was 245 nurses with 90.7% response rate. According to our findings, in 86 nurses the mean moral reasoning score was lower than the community average. Among 86 nurses, 66 nurses who willingness to participate in the study if they did not have a history of attending in workshops or nursing ethic courses in the past, were selected by simple random sampling method and assigned into three groups (two experimental groups and one control group) using block randomization methods.

### Sample size

based on the study of Borhani et al. [31], and using Altman nomogram with the confidence coefficient of 95%, the confidence interval of 1.96, type II error of 10% (1.63) and 90% power, the sample size was initially set at 17 subjects in each group which was raised to 22 subjects in view of the possibility of a 10% sample loss.

### Randomization

The subjects were included in the study by simple random sampling method and divided into three equal groups (n = 22) including two experimental groups (received nursing ethics education through lectures and group discussions methods) and a control group (did not received any nursing ethics training) using block randomization methods. To perform blocked randomization, the randomization code was generated by computer in permuted blocks of 6. Block randomization was performed using sealed envelope technique and computer-generated random numbers by Random Allocation Software © (RAS; Informer Technologies, Inc., Madrid, Spain).

### Intervention

Nursing ethics education lesson plan in both experimental groups was conducted in a one-day workshop for 8 h in order to get acquainted with the basics and principles of ethical decision-making and to acquire ethical reasoning skills. The details of training program implemented for the two intervention groups is available in Additional File 1. Education program via lectures method was conducted as a one-day symposium and the answers to questions of each scenario and the solutions to the ethical dilemmas presented in each scenario were provided by lecturer. Training for the group discussions was presented as a one-day workshop as a discussion and by giving predetermined scenarios, the participants were asked about ethical issues in the field of nursing. They made ethical decisions in the face of these issues and reasonably defended their decision, and participants criticized each other’s decisions. In summarizing the discussion between the participants about each scenario, the researcher based on scientific principles approved or rejected the participants’ decisions and was taught how to make ethical decisions, so that nurses at the end of the workshop could formulate ethical issues with critical reasoning and identify the correct moral decision.

The educational content for both experimental groups was the same. So, there was no risk of between‑group information leakage. The educational content included teaching the basics and principles definitions of ethics, the importance of nurses’ awareness of ethics, the importance of ethics to nursing, the ethical principles of nursing practice (including independence, secrecy, and accountability), professional ethics, and the approaches to ethical decision‑making, Kohlberg’s level of moral development and presenting ethical scenarios. To determine the validity of the program syllabus, the opinions of faculty members and members of the hospital ethics committee were used.

### Data collection and study instruments

Data were collected using four questionnaires including sociodemographic questionnaire, Nursing Dilemma Test (NDT), Moral Distress Scale (MDS) and Moral Sensitivity Questionnaire (MSQ). To determine the levels of moral reasoning, moral distress, and moral sensitivity of these nurses, the questionnaires were completed once by the participants. However, to examine and comparison the effect of training in ethical decision-making, the questionnaires were completed by the participants twice, pre- and post-intervention.

**Sociodemographic questionnaire;** The sociodemographic data questionnaire consisted of twelve questions about age (years), gender (male and female), marital status (married and single), work experience (years), work wards (general ward and intensive ward), position (head nurse, nurse, and in charge nurse), employment type (full time, part time and contract employees), shift work (fixed shift and rotation shift), overtime work (hours), awareness of code of ethics (completely, partly and never), awareness of Patients’ Rights (completely, partly and never) and attending to ethics course (yes and no).

**Nursing Dilemma Test (NDT);** The NDT was developed by Patricia Crisham in 1981 at the University of Minnesota through studying 130 nurses [32]. The NDT contains six scenarios on ethical dilemmas in nursing care, which includes (a) newborn with anomalies; (b) forcing medication; (c) adults’ requests to die; (d) new nurse orientation; (e) medication errors and (f) terminally ill adults. Each scenario consists of three sections; first section asks about the necessary action in case of a moral dilemma presented in the scenario and wants the answerer to mark one of the three options provided for each ethical dilemma, which the answer can be interpreted in three ways: correct, incorrect and unanswered. Second section is based on the Kohlberg’s Moral Development Theory and in this part six statements are presented which asked the participants are asked to choose the most important statement among these six and to put the statements in order of importance for themselves. Responses given in this part determined the scores of Nursing Principled thinking (NPT). NPT shows the importance attached to considering moral principles when making a moral decision in nursing. The lowest and highest NPT score from each scenario is 3 and 11. Thus, the range of total score for six scenarios is 18 to 66 and a higher score of NPT indicates a higher level of moral reasoning abilities. The third section assesses whether participants had previous experiences with a similar dilemma or not. A familiarity score between 6 and 17 shows that the participants are familiar with a similar dilemma, while a score falling within the 18–30 range reveals no familiarity with a similar dilemma. The Cronbach’s alpha coefficient for the Persian version of the NDT was reported 0.82 and 0.95 by Borhani et al. [33], and Zirak et al. [34], respectively.

**Moral Distress Scale (MDS);** The MDS was developed and validated by Atashzadeh et al. [35], in Iran, which assesses the severity of moral distress in ICU nurses. This tool contains 30 items that include three dimensions; inappropriate competencies and responsibilities (10 items), errors (11 items) and not respecting the ethics principles (9 items). The scoring system for this scale is based on four-point Likert scale (0 = none to 4 = very much). Each item received 0–4 points and the whole instrument had a score between 0 and 120. The moral distress score is obtained from the average total points of the items. Similarly, the score of each dimension is obtained from the average total points of the items of the same dimension. The moral distress score obtained from the whole scale was grouped into four categories (0–1 = low, 1.01–2 = average, 2.01–3 = high, 3.01–4 = very high). Thus, the obtained score ranged from low to very high of which the higher score indicates the existence of more moral tension. The Cronbach’s alpha coefficient for the total MDS score and each dimension that includes inappropriate competencies and responsibilities, errors and not respecting the ethics principles was reported 0.93, 0.93, 0.96, and 0.89 by Atashzadeh et al. [36], respectively.

**Moral Sensitivity Questionnaire (MSQ);** The MSQ was developed by Lutzen et al. [37], in Sweden and then was used in various countries, including Iran. This tool measures the ethical status of nurses when providing clinical services. The first questionnaire had 30 items, which reduced to 25 items during the process. The questionnaire is comprised of six subscales: respect the patient’s autonomy (questions 1, 10, 12), knowledge of how to communicate with the patient (questions 1, 2, 3, 4, 17), professional knowledge (questions 16, 24), experience of ethical problems and conflicts (questions 9, 11, 15), the application of moral concepts in moral decisions (questions 6, 8, 14, 18, 20) and integrity and benevolence (questions 5, 7, 19, 21, 22, 23, 25). It is scored based on five-point Likert scale (0 = no comment to 4 = totally agree). The overall score of this scale is between 0 and 100. The total scores between 0 and 50, 50–75 and 75–100 indicates low, moderate and high level of moral sensitivity, respectively. The reliability of the questionnaire in the US and in Korea was 0.76 and 0.78, respectively [38, 39]. In addition, the Cronbach’s alpha coefficient for the Persian version of the MSQ instrument was reported 0.80 and 0.81 by Izadi et al. [40], and Hassanpoor et al. [41], respectively.

### Statistical analysis

Categorical variables were described as frequency rates and percentages, and continuous variables were described using mean ± standard deviation (SD) values. Inferential statistics such as independent t-test and one-way analysis of variance (ANOVA) and Bonferroni post hoc test were used to examine the association of NDT, MD and MS and their dimensions’ mean scores with sociodemographic variables. Chi-square (χ2) or Fisher’s exact tests were used for comparing sociodemographic characteristics as categorical proportions with three groups of study. One-way ANCOVA (analysis of covariance) with repeated measures (RMANOVA) was used to assess the time trend and group interaction effects on the mean scores of NDT, MD and MS pre-and post-intervention in the three study groups. Both unadjusted and adjusted (adjusting for based on age group, gender, marital status, work experience, wards, shift work and overtime) repeated measures ANOVA were assessed. Multiple Bonferroni post hoc test was used to explore differences between pairwise groups in means of questionnaires scores. Univariate and multivariate binary logistic regression were used to evaluate the association of sociodemographic characteristic with the scores of moral reasoning, moral distress and moral sensitivity of 245 nurses. In addition, unadjusted and adjusted binary logistic regression analysis were used to assessed the association between three groups of study with the scores of moral reasoning, moral distress and moral sensitivity of 66 nurses after intervention. Associations in regression analysis were reported using the odds ratio (OR) and 95% confidence intervals. (CI) GraphPad Prism 9© (GraphPad Software Inc., La Jolla, CA) was used for forest plot of logistic regression analysis to show the association of parameters. All analyses were conducted using SPSS software (ver.21) (SPSS Inc. IL, Chicago, USA) and in all analyses, a two-tailed P-value of < 0.05 was considered significant.

## Results

### Levels of moral reasoning, moral distress and moral sensitivity

A total of 245 nurses have completed the all questionnaires. Distribution the mean total scores of nursing dilemma test, moral distress and moral sensitivity according to sociodemographic characteristics of 245 nurses are available in Additional File 2 Table S1. In nursing dilemma test, the mean total score of nursing principled thinking (NPT) (section B) and familiarity (section C) of the nurses were 40.80 ± 6.71 and 13.55 ± 4.09, respectively. The level of moral reasoning of 39 (15.9%), 187 (76.3%) and 19 (7.8%) of the nurses was pre-conventional, conventional and post-conventional, respectively. The mean total NPT score among the single nurses was significantly higher than married nurses (43.13 ± 7.60 vs. 40.45 ± 6.52, P = 0.035). In terms of familiarity, the results showed that the majority of nurses were familiar with similar dilemmas (n = 192, 78.4%). No significant differences were observed between familiarity score and sociodemographic characteristics (P > 0.05).

The mean total score of moral distress in nurses was 60.66 ± 26.23, which was slightly higher than average (Additional File 2 Table S1). Additionally, the findings revealed mean score of MD in “inappropriate competencies and responsibilities” dimension was 18.29 ± 9.44, in “errors” dimension, was 23.12 ± 10.35 and in “not respecting the ethics principles” dimension was 19.25 ± 9.05 (Additional File 2 Table S2). There was no statistically significant difference between the sociodemographic factors and moral distress and its dimensions.

The mean score of total moral sensitivity in the nurses was 63.78 ± 10.47, which indicates the moderate level of moral sensitivity in them (Additional File 2 Table S1). No significance differences were observed between moral sensitivity and sociodemographic characteristics. Distribution the scores of moral sensitivity’s dimensions according to sociodemographic characteristics are presented in Additional File 2 Table S3. The findings revealed mean score of MS in “respect the patient’s autonomy” dimension was 9.73 ± 1.99, in “knowledge of how to communicate with the patient” dimension, was 15.88 ± 3.76, in “professional knowledge” dimension was 3.65 ± 1.73, in “experience of ethical problems and conflicts” dimension was 8.48 ± 1.96, in “the application of moral concepts in moral decisions” dimension was 11.62 ± 3.04 and in “integrity and benevolence” dimension was 15.81 ± 3.70. In “respect the patient’s autonomy” dimension was observed that female nurses’ score was significantly higher than male (10.08 ± 17.33 vs. 8.96 ± 2.29, P = 0.001), contract employee status nurses had higher score than part time nurses (10.30 ± 1.47 vs. 8.17 ± 2.91, P = 0.005) and nurses with lower overtime work (≤ 60 h) had higher score than the nurses with more than 60 h overtime work (10.03 ± 1.85 vs. 9.37 ± 2.10, P = 0.009). According to the “knowledge of how to communicate with the patient” dimension, female nurses’ score was significantly higher than male (16.45 ± 3.80 vs. 14.64 ± 3.41, P = 0.001), nurses with higher work experience (< 15 years) had higher score than the nurses with lower work experience (≤ 15 years) (16.43 ± 4.14 vs. 15.37 ± 3.31, P = 0.027), employee status of nurses had impact on the score of this dimension as contract employee nurses had higher score than the full time (16.89 ± 4.15 vs. 15.36 ± 3.37, P = 0.05) and part time nurses (16.89 ± 4.15 vs. 14.17 ± 3.43, P = 0.006), the score was higher in the nurses with lower overtime work than that higher overtime work (16.34 ± 4.03 vs. 15.34 ± 3.36, P = 0.038). The score of “professional knowledge” dimension in married nurses was significantly higher than single nurses (3.74 ± 1.73 vs. 3.06 ± 1.62, P = 0.040) and nurses working in ICU than those working in general wards (3.97 ± 1.74 vs. 3.07 ± 1.67, P = 0.002). The score of experience of ethical problems and conflicts dimension in female nurses was significantly higher than the male nurses (8.71 ± 1.81 vs. 7.99 ± 2.20, P = 0.007). In addition, the significantly higher score in “the application of moral concepts in moral decisions” dimension was observed in nurses with fixed shiftwork than nurses with rotation shiftwork (12.01 ± 2.82 vs. 11.02 ± 3.26, P = 0.012).

### Results from section A of NDT

The data obtained from section A of each scenario of NDT are showed in Additional File 2 Table S4. According to the results, more than half of the nurses (65.3%) were in favor of resuscitation of a newborn with abnormalities, 24.9% supported administering medication against the will of the patient while, and 4.1% of them remained undecided. As for the third scenario, the majority of the nurses (93.5%) stated that they would provide respiratory support although a competent adult patient requested to die. Nearly one third of the nurses (33.9%) stated that time should be set aside for the orientation of new nurses, and 8.6% of them remaining undecided. A great majority of the nurses (90.6%) stated that medication errors must be informed. The last scenario presented a dilemma about a terminally ill adults and fewer than half of the nurses (44.45%) thought that patients’ questions must be answered and 15.5% remained undecided.

### Binary logistic regression findings

**Fig. 1:**
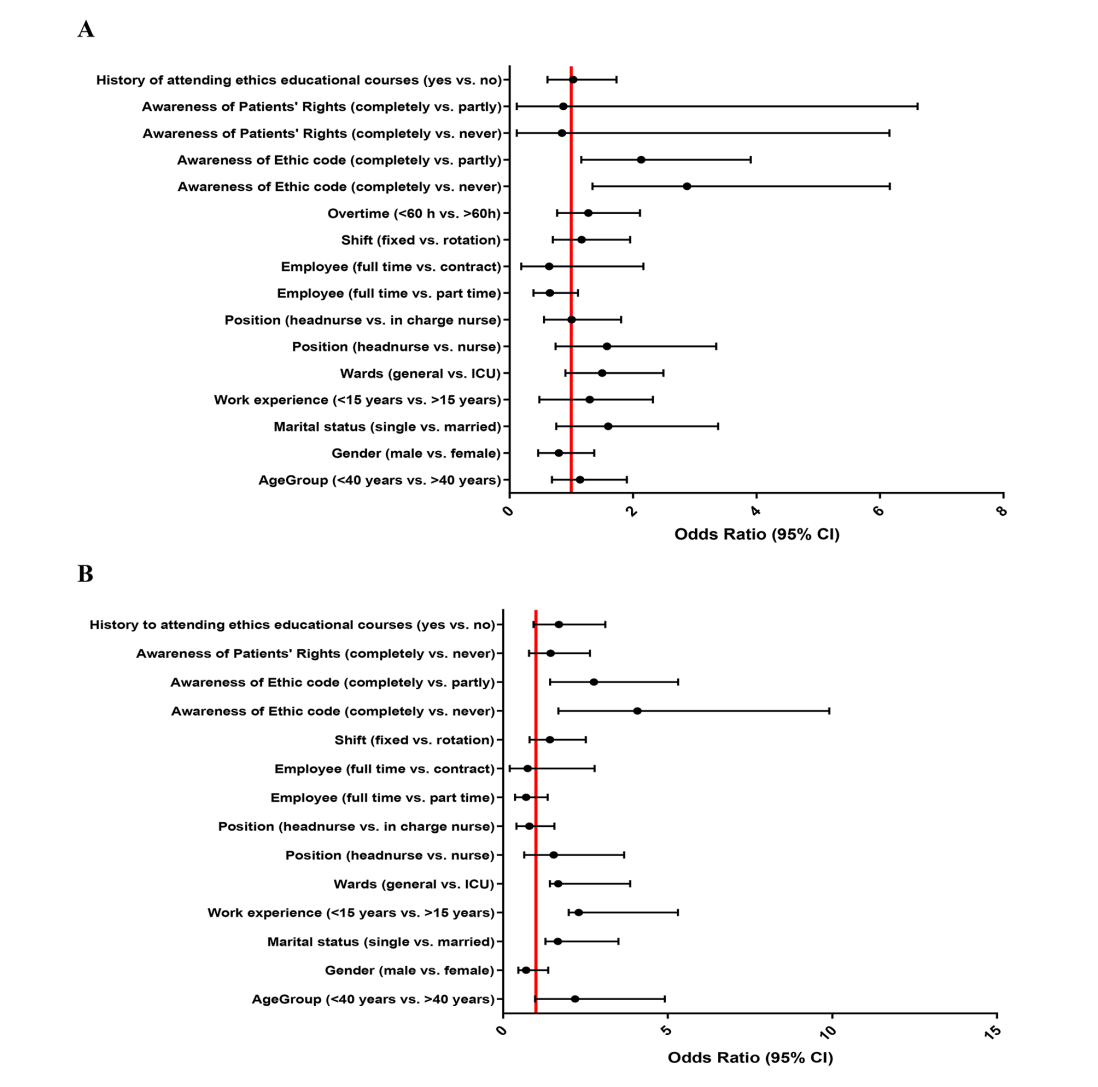
Forest plot of (**A**) univariate and (**B**) multivariate binary logistic regression analysis to show the association of sociodemographic characteristic with the section B (NP score ≤ 41 vs. >41) of the Nursing Dilemma Test

Univariate and multivariate binary logistic regression analysis to evaluate the association of sociodemographic characteristic with NPT score (section B of NDT) are presented in Fig. 1A and B. In multivariate regression analysis, the NPT score was significantly increased by single status (OR: 1.66, 95% CI: 1.289–3.506, P = 0.023), lower (≤ 15 years) work experience (OR: 2.297, 95%CI: 1.993–5.314, P = 0.042), working in general wards (OR: 1.677, 95%CI: 1.023–3.858, P = 0.045) and completely awareness of code of ethics than partly (OR: 2.757, 95%CI: 1.43–5.316, P = 0.002) and never (OR: 4.08, 95%CI: 1.68–9.909, P = 0.001) awareness. Furthermore, Univariate and multivariate binary logistic regression analysis to evaluate the association of sociodemographic characteristic with familiarity, MD and MS scores are available in Additional File 2 Table S5–S7. However, no significant association was observed between the factors and scores of familiarity, MD and MS.

### Characteristics of nurses in the second phase of study (n = 66)

**Fig. 2:**
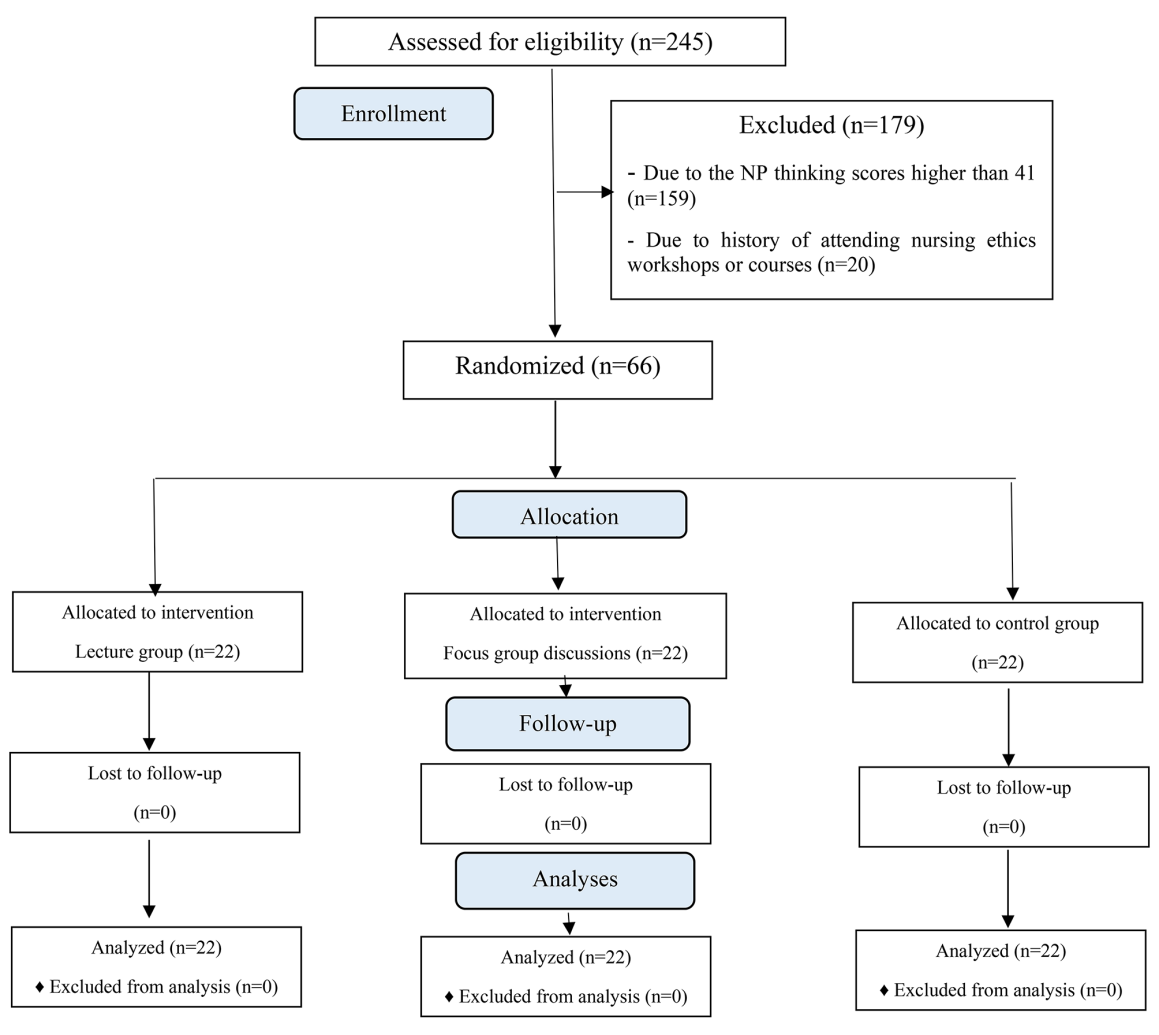
CONSORT flow diagram

The CONSORT flow diagram of study population in the second phase of study is presented in Fig. 2. Of the 245 nurses who participated in the first phase of the study, 66 nurses with NPT scores below the community average, no history of attending the nursing ethics education, and willingness to participate were selected for the second phase. These 66 nurses randomly assigned into three equal groups (n = 22) including two experimental groups (lectures method and group discussions methods) and one control group. Mean age of the patients in the lecture group, group discussion, and control group were 37.32 ± 7.93, 40.05 ± 5.63, and 37.32 ± 6.41 years, respectively, with female predominance of 77.3%, 63.6%, and 68.2%, respectively, in the three groups of study. According to post hoc Tukey test, the mean age of almond group was significantly higher than the mean age of the patients in the lavender group (63.19 ± 9.07 vs. 56.92 ± 9.12, P = 0.016). Also, in terms of marital status, the significant statistically difference was observed between the three groups of study (P < 0.001). However, there was no statistically significant difference between the three groups regarding in the gender (P = 0.729) and qualification (P = 0.078) The results indicated that there was no significant difference between the three groups in terms of the demographic variables, including age (P = 0.555), gender (P = 0.605), marital status (P = 0.288), work experience (P = 0.832), ward of working (P = 0.650), position (P = 0.528), employment types (P = 0.136), shift working (P = 0.299), overtime working (P = 0.785) and awareness of patients’ rights (P = 0.683) (Table 1). However, awareness of ethical code was significantly higher in the group discussions than the other groups (P = 0.002).

**Table 1:** Sociodemographic characteristics of the participants in three groups of study (n = 66)

Sociodemographic characteristics		Total (n = 66)	Lecture group (n = 22)	Discussion group (n = 22)	Control group (n = 22)	P-value
Gender	Male	20 (30.3)	5 (22.7)	8 (36.4)	7 (31.8)	0.605
	Female	46 (69.7)	17 (77.3)	14 (63.6)	15 (68.2)	
Age (year)	≤ 40	42 (63.6)	15 (68.2)	12 (54.5)	15 (68.2)	0.555
	> 40	24 (36.4)	7 (31.8)	10 (45.5)	7 (31.8)	
Marital status	Single	13 (19.7)	6 (27.3)	5 (22.7)	2 (9.1)	0.288
	Married	53 (80.3)	16 (72.2)	17 (77.3)	20 (90.9)	
Work experience	≤ 15	36 (54.5)	11 (50)	12 (54.5)	13 (59.1)	0.832
(year)	> 15	30 (45.5)	11 (50)	10 (45.5)	9 (40.9)	
Ward of	General	29 (43.9)	11 (50)	10 (45.5)	8 (36.4)	0.650
working	ICU	37 (56.1)	11 (50)	12 (54.5)	14 (63.6)	
Position	Head nurse	10 (15.2)	4 (18.2)	4 (18.2)	2 (9.1)	0.528
	In charge nurse	19 (28.8)	6 (27.3)	4 (18.2)	9 (40.9)	
	Nurse	37 (56.1)	12 (54.5)	14 (63.6)	11 (50)	
Employment	Full time	39 (59.1)	11 (50)	11 (50)	17 (77.3)	0.136
types	Part time	4 (6.1)	3 (13.6)	1 (4.5)	0	
	Contract employees	23 (34.8)	8 (36.4)	10 (45.5)	5 (22.7)	
Shift working	Fixed shift	40 (60.6)	16 (72.7)	13 (59.1)	11 (50)	0.299
	Rotation shift	26 (39.4)	6 (27.3)	9 (40.9)	11 (50)	
Overtime work	≤ 60	34 (51.5)	10 (45.5)	12 (54.5)	12 (54.5)	0.785
(hours)	> 60	32 (48.5)	12 (54.5)	10 (45.5)	10 (45.5)	
Awareness of	Completely	12 (18.2)	4 (18.2)	8 (36.4)	0	0.002*
Ethical code	Partly	41 (62.1)	14 (63.6)	14 (63.6)	13 (59.1)	
	Never	13 (19.7)	4 (18.2)	0	9 (40.9)	
Awareness of	Completely	49 (74.2)	15 (68.2)	17 (77.3)	17 (77.3)	0.683
Patients’ Rights	Partly	16 (24.2)	6 (27.3)	5 (22.7)	5 (22.7)	
	Never	1 (1.5)	1 (4.5)	0	0	

*P < 0.05 considered as significant

### Comparison of pre- and post-intervention scores

Comparison of pre- and post-intervention scores of nursing dilemma, moral distress and moral sensitivity between three groups are presented in Table 2. The results showed no significant differences between the three groups in terms of their NPT (P = 0.838), familiarity (P = 0.640), moral distress (P = 0.931) and moral sensitivity (P = 0.159) scores in pre-intervention. At the beginning of the study, the mean NPT scores did not differ significantly between the three groups (P = 0.838), however, after the intervention, this mean score increased significantly in the both experimental groups compared to the control group (P < 0.001). Also, between the two experimental groups, the improved NPT score after the intervention in the discussion group was significantly higher than the lecture group (52.50 ± 2.44 vs. 44.64 ± 4.70, P < 0.001). However, the differences between the mean scores of nurses’ familiarity after the intervention in the three studied groups was not significant (P = 0.997). The mean score of the post-intervention moral sensitivity in the discussion group was significantly higher than the lecture (76.50 ± 11.52 vs. 61.55 ± 11.57, P < 0.001) and control groups (76.50 ± 11.52 vs. 64.27 ± 9.45, P < 0.001). In terms of moral distress, the difference between the mean total scores and its dimensions after the intervention in the three studied groups was not significant, but in the dimension of “not respecting the ethics principles” in pre- and post-intervention, a significant decrease was observed between the two intervention groups after intervention. These differences in the lecture and discussion groups were (21.91 ± 9.59 to 17.36 ± 7.75, P = 0.020) and (17.27 ± 8.54 to 12.59 ± 4.82, P = 0.017), respectively. In addition, in this dimension significantly differed was observed between the discussion group and control group (12.59 ± 4.82 vs. 20.09 ± 10.07, P = 0.007).

**Table 2:** Comparison of pre- and post-intervention scores of nursing dilemma, moral distress and moral sensitivity between three groups

Parameters	Times	Lecture group (n = 22)	Discussioin group (n = 22)	Control group (n = 22)	P-value ***	P-value ****
Nursing dilemma test (NDT)						
NP score	Pre-intervention	36.09 ± 5.28	36.18 ± 3.72	35.45 ± 4.09	0.838	< 0.001*
	Post-intervention	44.64 ± 4.70	52.50 ± 2.44	35.36 ± 4.03	< 0.001*	
	P-value**	< 0.001*	< 0.001*	0.936		
Familiarity score	Pre-intervention	14.91 ± 3.41	14.50 ± 3.76	13.82 ± 4.33	0.640	0.811
	Post-intervention	13.68 ± 3.63	13.46 ± 3.67	13.73 ± 4.39	0.997	
	P-value*	0.203	0.445	0.943		
Moral Sensitivity Questionnaire (MSQ)						
Total moral sensitivity score	Pre-intervention	60.36 ± 11.68	59.23 ± 11.43	65.55 ± 11.34	0.159	0.010*
	Post-intervention	61.55 ± 11.57	76.50 ± 11.52	64.27 ± 9.45	< 0.001*	
	P-value*	0.742	< 0.001*	0.689		
Moral Distress Scale (MDS)						
Total Moral distress score	Pre-intervention	60.23 ± 21.34	57.32 ± 26.93	58.09 ± 30.32	0.931	0.337
	Post-intervention	53.41 ± 21.34	44.73 ± 17.24	61.32 ± 31.93	0.085	
	P-value*	0.176	0.104	0.754		
Moral distress’s dimensions						
Inappropriate competencies	Pre-intervention	17.32 ± 7.18	17.27 ± 7.85	15.82 ± 10.90	0.814	0.651
and responsibilities	Post-intervention	16.77 ± 7.86	14.68 ± 8.84	16.86 ± 11.11	0.684	
	P-value*	0.806	0.381	0.785		
Errors	Pre-intervention	23.05 ± 9.79	22.77 ± 12.30	23.09 ± 12.11	0.995	0.316
	Post-intervention	18.82 ± 9.11	17.45 ± 6.78	24.36 ± 12.78	0.056	
	P-value*	0.059	0.117	0.739		
Not respecting the ethics	Pre-intervention	21.91 ± 9.59	17.27 ± 8.54	19.18 ± 9.45	0.252	0.175
principles	Post-intervention	17.36 ± 7.75	12.59 ± 4.82	20.09 ± 10.07	0.009*	
	P-value*	0.020*	0.017*	0.771		

Data are presented as mean ± SD; * P < 0.05 considered as significant, ** Obtained from paired t-test (within-group differences); *** Obtained from unadjusted one-way ANCOVA (analysis of covariance) with repeated measures (RMANOVA) (between-group differences); **** Obtained from adjusted (based on age group, gender, marital status, work experience, wards, shift work and overtime) one-way ANCOVA (analysis of covariance) with repeated measures (between-group differences)

### Findings of regression analysis between groups

**Fig. 3:**
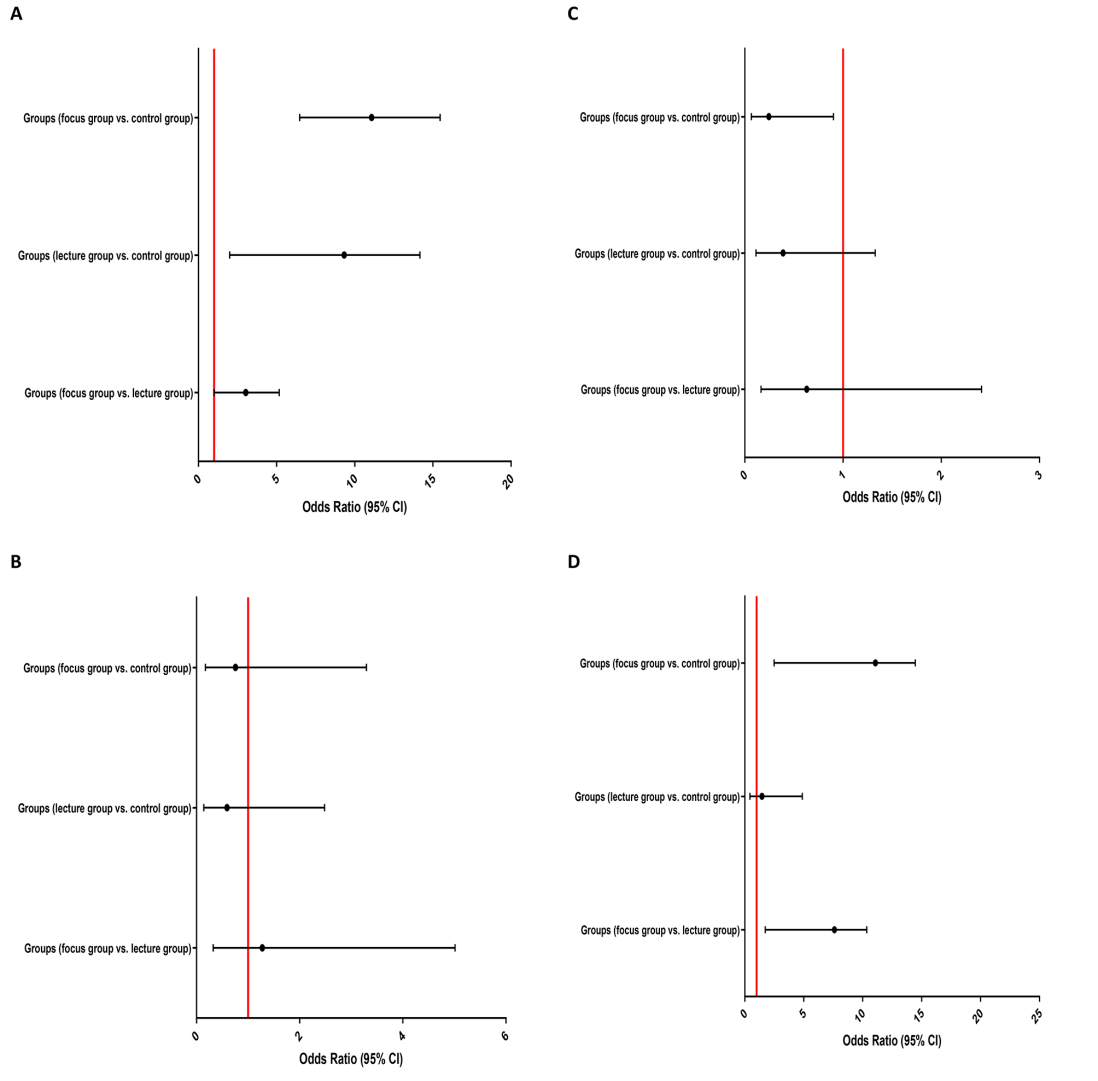
Forest plot of unadjusted binary logistic regression analysis to evaluate the association between three groups of study with the (**A**) NPT score (≤ 50 vs. >50), (**B**) familiarity score (≤ 18 vs. >18), (**C**) moral distress score (≤ 58 vs. >58) and (**D**) moral sensitivity score (≤ 75 vs. >75)

**Fig. 4:**
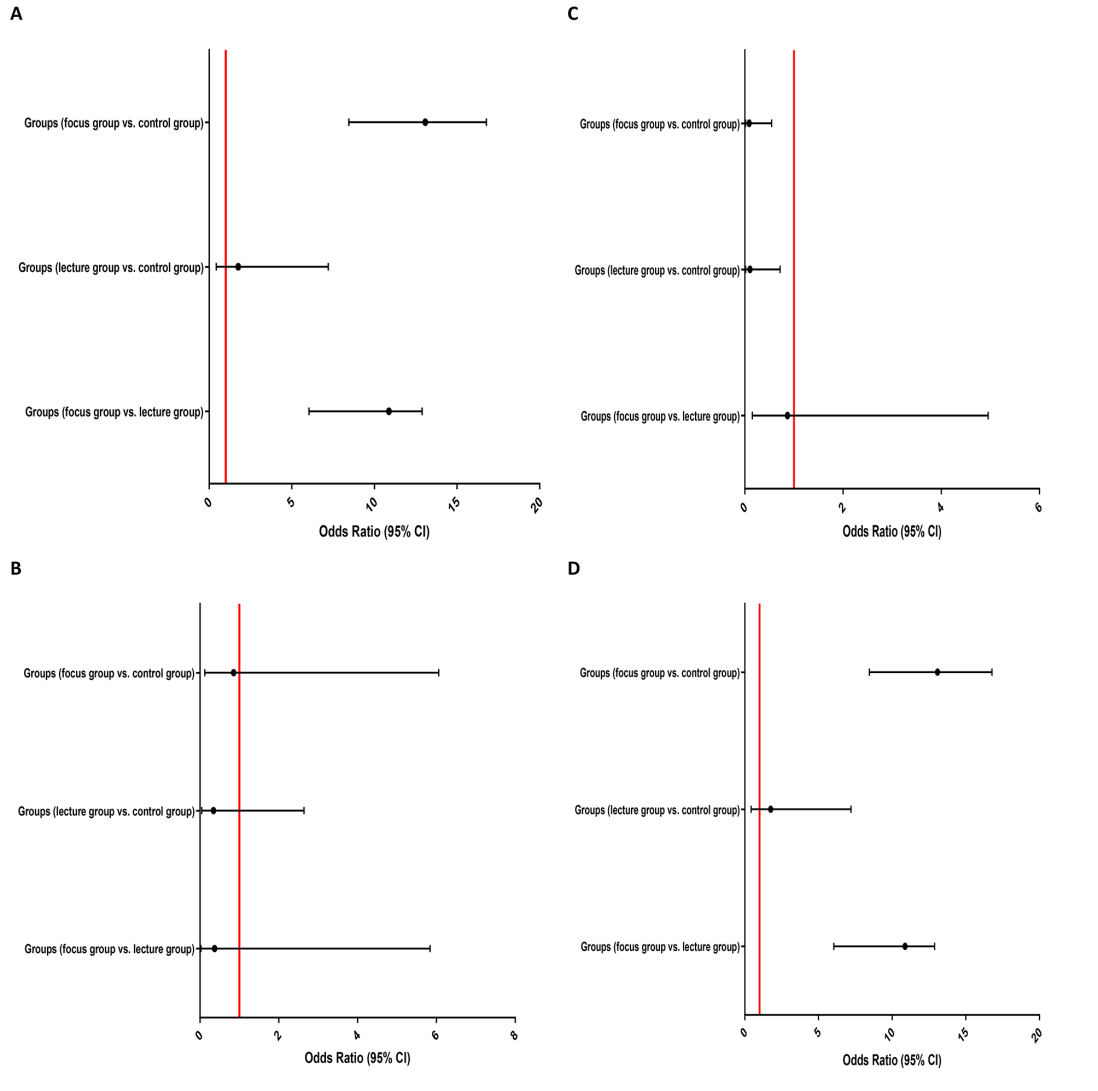
Forest plot of adjusted binary logistic regression analysis to evaluate the association between three groups of study with the (**A**) NPT score (≤ 50 vs. >50), (**B**) familiarity score (≤ 18 vs. >18), (**C**) moral distress score (≤ 58 vs. >58) and (**D**) moral sensitivity score (≤ 75 vs. >75)

Unadjusted and adjusted binary logistic regression analysis to evaluate the association between three groups of study with the NPT score, familiarity score, moral distress score and moral sensitivity score are presented in Figs. 3 and 4. Adjusted regression analysis showed that the probability of increasing the nursing principle thinking (NPT) score through discussion training was significantly higher than lecture (OR: 13.078, 95% CI: 3.238–15.954, P = 0.008), as well as lecture (OR: 14.329, 95% CI: 16.171–2.005, P < 0.001) and discussion groups compared to the control group (OR: 18.01, 95% CI: 22.15–5.834, P < 0.001). The possibility of increasing moral sensitivity score through discussion training was significantly higher than lecture (OR: 10.874, 95%CI: 6.043–12.886, P = 0.005) and control group (OR: 13.077, 95%CI: 8.454–16.774, P = 0.002). Moreover, the moral distress score was significantly reduced only in the trained group compared to the control, and no significant difference was observed between the experimental groups; lecture group vs. control group (OR: 0.105, 95% CI: 0.015–0.717, P = 0.021) and discussion group vs. control group (OR: 0.089, 95% CI: 0.015–0.547, P = 0.009). Details of all regression analyzes are available in the Additional File 2 Table S8–S11.

## Discussion

The results of this study showed that the level of moral reasoning, moral sensitivity and moral distress in the nurses compared to previous studies and the level of average community, were low, medium and high, respectively. The results of the nurses’ responses to the questions of part A of the NDT test showed that many of them are familiar with these problems, which is a confirmation of the findings of part C of the NDT test. In this study, familiarity scores of the majority nurses indicated that they were familiar with ethical dilemmas. Familiarity of the nurses with ethical dilemmas is considered to affect their decision making for ethical problems positively [34, 42]. However, ethical decision-making of the nurses may be affected by the several factors such as policies and expectations of the institutions where they work, feeling of mastery of the medical profession, stressful environment, patient complex conditions, and the communication with patients that instead of following the rules, they listen to their inner voice [43, 44].

The present study found that the mean NPT scores of nurses were slightly lower than the average level (40.80 ± 6.71). In previous studies by Zirak et al. [34], Borhani et al. [45], and Ham et al. [46], the NPT scores reported 46.67 ± 6.7, 42.16 ± 5.8 and 51.5 ± 7.9, respectively. These differences could be due to the attitudes and beliefs of nurses can be influenced by several factors, including rules of workplace and regulations, level of education, cultural, social, political, religious, and clinical experiences of nursing [47]. According to multivariate regression analysis, single status, less work experience (≤ 15 years), work in the general wards, and awareness of the code of ethics were found to be associated with a higher score of NPT. The results of second phase of study showed that the post-intervention mean score of the NPT scale was significantly higher in both experimental groups than the control group. The majority of participants (76.3%) were at the conventional level of moral reasoning, and according to Kohlberg’s theory, the basis of their moral reasoning is to adhere to the rules of the organization, to be in harmony with the community, and to show oneself well in the eyes of others. The low number of nurses (7.8%) with post-conventional level of ethical reasoning can be due to the overemphasis on the authorities to unreasonably follow the rules of the organization and also the complexity of ethical decision-making in clinical settings, which reduces nurses to the customary level of Kohlberg’s moral development stages. Nurses try to have arguments in line with other colleagues that are approved by colleagues and the institute [4, 5]. Therefore, after completing the training course with the main purpose of familiarizing nurses with the concepts of professional ethics, the principles of nursing ethics and ethical conflicts and challenges in clinical care and acquiring ethical decision-making skills, NPT scores were increase among learners.

In this study, total mean score of moral distress include “inappropriate competencies and responsibilities” and ‘‘errors’’ dimensions were high. However, moral distress in ‘‘not respecting the ethics principles’’ dimension was moderate. The highest moral distress was related to ‘‘errors’’ dimension, which was consistence with a study by Atashzadeh-Shoorideh et al. [36]. No significant association was found between moral distress levels of the nurses and their sociodemographic variables. The lack of connection between moral distress and sociodemographic characteristics can mean that all nurses, regardless of age, gender, work experience, ward of works, shift work, and etc., experience moral distress. According to evidence more than half of nurses experience moral distress in their work environment [48, 49]. Provide education and training to reduce moral distress and its impact on nurses is very important. Although the results of the second phase of the study showed that the training did not have much effect in reducing the moral distress scores of the nurses and only dimension “not respecting the ethics principles” there was a significant reduction. Moral distress may be disrupting the moral decision-making process of achieving care system objectives and consequently have an adverse effect on the health pattern of the society. On the other, it can create mental and physical problems for nurses, which may influence on occupational satisfaction and their willingness to remain in the profession, and eventually the quality of care [35, 50]. It seems that training alone is not enough to reduce the two dimensions “inappropriate competences and responsibilities” and “error” of moral distress. Therefore, it is essential to create a safe and supportive environment for nurses to express their feelings and concerns without fear of punishment. Creating a culture of open communication and collaboration between nurses and other members of the health care team. Morover, increasing rewards for nurses who manage moral distress, creating support systems for nurses dealing with moral distress, providing resources for nurses to make decisions, as well as recognizing and address systemic issues that may be contributing to moral distress.

Current study showed that the majority of nurses have moderate moral sensitivity, which was consistent with some previous studies [17, 51]. Because nurses deal with serious situations in patient care that require ethical ability to make appropriate decisions and actions, they need to be sensitive and familiar with ethical issues related to their profession. In this study, there was no significant relationship between sociodemographic characteristics with moral sensitivity, which is consistent with the study of Hassanpoor et al. [41]. Therefore, it can be concluded that all nurses, regardless of individual and professional conditions, have moderate moral sensitivity, which should be identified as limiting factors of moral sensitivity. Therefore, moderate moral sensitivity of nurses in this study can be due to the “patient-related”, “environmental” and “managerial”, the most important obstacle related to patients includes the patient’s lack of knowledge about the nurse’s job description and the most important environmental obstacle of the crowded ward [52]. The findings of this study showed that the moral sensitivity in nurses was improved with training in ethical decision-making program.

In line with previous research [53–55], the findings of this study showed that moral reasoning decreases with increasing nurses’ work experience. The reasons for this can be crowded work shifts, burnout, job dissatisfaction, stressful and diverse work environment that make nurses unmotivated by the challenges of clinical ethics. On the other hand, with increasing work experience, nurses ‘commitment to the institution increases and nurses may prefer organizational interests to patients’ rights [56]. The findings from this research confirmed that ethical behavior is more influenced by the ethics educational program rather than sociodemographic factors. The results also indicated that the group discussions‑based ethics education be more effective in improving the abilities of moral reasoning and moral sensitivity than lecture method. In teaching via lecture method, a large amount of information is provided to nurses in a short period of time. Additionally, in this method, participants are mostly passive, and hence, not all of their problem-solving, decision-making and analytical abilities can be improved. Although the scores of nurses’ ethical reasoning increased with this method compared to the control group, but it was significantly lower to compare with the group discussions. Therefore, it seems that in this method, nurses still have problems and low self-confidence for decision‑making in difficult situations and knowledge use in practice. While the results showed that ethics education based on group discussions significantly improved nurses’ moral reasoning and moral sensitivity. The positive effects of group discussions can be attributed to the active involvement of nurses in the process of learning, their group discussions on learning materials, and their exposure to realistic or simulated situations and cases [57].

### Limitations

The present study has some limitations such as the first phase of study was carried out as descriptive study in single-center hospital, and data was collected using self-reported questionnaires. There may be a possibility of bias and exaggeration of scores. The time interval between the intervention and post-test outcome assessment was also short, and thus, the study provided no information about the long‑term effects of education program on moral reasoning, sensitivity and distress. Ultimately, it should be considered that the impact of one session training on nurses through discussion was very high (Hedge’s g effect size), a possible reason for this extreme finding is that the study focused on training low performers. Therefore, the effects that were found would probably be significantly lower for nurses with lesser development needs. For these reasons, the generalization of the findings is limited and also the results should be interpreted with caution. Nevertheless, the findings of the study would be helpful for nurse educators and policy makers to develop continuous training in ethical decision-making to improve nurses’ awareness and understanding of the importance of moral decision-making process and improve the quality of care.

## Conclusion

According to the results, moral reasoning, moral sensitivity and moral distress in the nurses compared to previous studies and the level of average community, were low, medium and high, respectively. In addition, single status, less work experience, work in the general wards, and awareness of the code of ethics were found to be associated with a higher ability to moral reasoning. While, there was no significant relationship between moral sensitivity and moral distress with any of the demographic characteristics of nurses. The findings of the randomized clinical trial phase of this study indicate that training ethical decision-making through group discussions is an effective strategy for improving moral reasoning and moral sensitivity in nurses, but has no effect on moral distress. Therefore, it can be said that all nurses at any level of sensitivity and moral reasoning experience moral stress that nursing managers should reduce the cause of stress. Therefore, it seems that the all nurses with any sociodemographic characteristics and also at any level of moral reasoning and moral sensitivity, experience moral distress that the policy makers should be find the strategy to reduce it.

### Electronic supplementary material

Below is the link to the electronic supplementary material.

Additional File 1: Educational program for the intervention group (lectures and group discussion).

Additional File 2: Table s1–Table s11.

## Data Availability

The data that support the findings of this study are available from the corresponding author upon reasonable request.

## Declarations

**Ethics approval and consent to participate**.

The present study was approved by the Ethics Committee of Baqiyatallah University of Medical Sciences, Tehran, Iran, under code IR.BMSU.REC.1394.145. This clinical trial study was registered in Iranian Registry of Clinical Trials under code (IRCT2015122116163N5) in 02/07/2016. Written informed consent was obtained from all participants. This study was performed and reported in accordance with the recommendations of the Consolidated Standards of Reporting Trials (CONSORT) statement and the Declaration of Helsinki of the World Medical Association.

**Consent for publication**.

## Abbreviations

NDT: Nursing Dilemma Test
MDS: Moral Distress Scale
MSQ: Moral Sensitivity Questionnaire
OR: Odds Ratio
GD: Group Discussions
