# Supplementary material for: The effect and comparison of training in ethical decision-making through lectures and group discussions on moral reasoning, moral distress and moral sensitivity in nurses: a clinical randomized controlled trial
Source: BMC Med Ethics. 2023 Aug 4;24:58. doi: 10.1186/s12910-023-00938-5 (PMC10403849; doi:10.1186/s12910-023-00938-5)
Supplement: Supplementary file 1 — Additional File 1: Educational program for the intervention group (lectures and group discussion). [file 12910_2023_938_MOESM1_ESM.docx]

- **Educational program for the intervention group in the form of lectures:**
- Main goal: to introduce nurses to the concepts of professional ethics, the principles of nursing ethics, and ethical conflicts and challenges in clinical care and to acquire ethical decision-making skills.
- Specific goals:

At the end of this training course, nurses are expected to:

- Explain the terms and concepts of ethics, professional ethics and ethics in nursing.

- Explain ethical theories, ethical philosophy and principles of bioethics and their applications in nursing.

- To be morally sensitive to ethical issues and dilemmas at the bedside.

- To explain the types of ethical conflicts and challenges and their causes in the clinical environment.

- To acquire the skill of ethical decision-making in nursing care

- **Training content table:**

| **No** | **Teaching title** | **Hours**  **(min)** | **Teaching methods** |
| --- | --- | --- | --- |
| 1 | Expressing learning goals and activities and reviewing the definitions, concepts and major topics of professional and creative nursing ethics | 45 | lecture |
| 2 | Ethical theories, principles of bioethics and their applications in nursing | 90 | lecture |
| 3 | Types and causes of ethical conflicts and challenges in the clinical environment | 90 | lecture |
| 4 | Ethical decision criteria and effective factors in ethical decision making | 45 | lecture |
| 5 | Ethical decision-making steps and principles of ethical decision making | 90 | lecture |
| 6 | The process of ethical action based on the "Rest" model | 60 | lecture |
| 7 | Kohlberg's stages of moral development | 60 | lecture |
| 8 | Question and answer | 60 | lecture |

- **Teaching methods:** lectures using slides and questions and answers
- **Educational program for the intervention group in the form of group discussion:**
- Main goal: to introduce nurses to the concepts of professional ethics, the principles of nursing ethics, and ethical conflicts and challenges in clinical care and to acquire ethical decision-making skills.
- Specific goals:

At the end of this training course, nurses are expected to:

- Explain the terms and concepts of ethics, professional ethics and ethics in nursing.

- Explain ethical theories, ethical philosophy and principles of bioethics and their applications in nursing.

- To be morally sensitive to ethical issues and dilemmas at the bedside.

- To explain the types of ethical conflicts and challenges and their causes in the clinical environment.

- To acquire the skill of ethical decision-making in nursing care

- **Training content table:**

| **No** | **Teaching title** | **Hours**  **(min)** | **Teaching methods** |
| --- | --- | --- | --- |
| 1 | Expressing learning goals and activities and reviewing the definitions, concepts and major topics of professional and creative nursing ethics | 45 | lecture |
| 2 | Ethical theories, principles of bioethics and their applications in nursing | 90 | lecture |
| 3 | Identifying the types of conflicts and ethical challenges in the clinical environment and sharing the experiences of nurses | 60 | Brainstorm |
| 4 | Expressing the experiences of nurses in facing ethical conflicts and challenges in the hospital environment and criticizing their decisions and performance | 120 | Group discussion |
| 5 | How learners behave ethically in the face of ethical scenarios based on the "Rest" model | 60 | Group discussion |
| 6 | The basis of learners' moral reasoning based on Kohlberg's stages of moral development | 120 | Group discussion |

- **Teaching methods:** lectures using slides, brainstorm and group discussion
- The most important challenges and conflicts identified in the brainstorming that nurses were morally sensitive to are as follows, and in a group discussion based on the nurses' own experiences, how to face these challenges was examined and criticized, and based on the "Rest" model, in nurses, the ratio Ethical sensitivity was created to these issues, how to reason rationally and make judgments about these challenges was practiced, and a desire to act morally was created in them.

• Colleagues who do not fulfill their duties and do less work

• Colleagues who have poor clinical performance and skills

• Error in diagnosis or doctor's order

• Conflicts between patients' wishes and hospital policies

• Caring for a patient who has no hope of treatment and is suffering from severe pain and wants to die

• Trying to care for and preserve the life of a newborn who has a severe birth defect

• Customized patient care

• Conflict between the interests of the organization and colleagues with the interests of the patient

- In the continuation of the group discussion sessions, six scenarios were given to the participants as follows and their decisions were criticized and analyzed based on Kohlberg's stages of moral development:

1. An 85-year-old man with advanced pancreatic cancer has been admitted to the intensive care unit. The nurse was preparing him for surgery, when he stated that no additional efforts should be made to prolong his life. After surgery, he was returned to the intensive care unit in a coma. During the night shift, the patient suffered a cardiac and respiratory arrest, the nurse immediately prepared the resuscitation equipment, but was in doubt whether to resuscitate the patient or not. If you were in his place, what would you do and why?

2. A baby boy was suctioned at the beginning of his birth due to aspiration of meconium, and the attending physician decided to insert a nasogastric tube, but the tube could not be passed. Finally, this tube was inserted, but the esophagus was torn during these attempts, so the baby is in mild respiratory distress. was transferred to the neonatal intensive care unit, this incident caused the infant, who could have been discharged with simple measures and less hospitalization days, to be hospitalized in the neonatal intensive care unit for 19 days, and the heavy cost of treatment was also imposed on the infant's family became. How do you act as a nurse in observing this incident and why?

3. I was working as the evening and night shift manager of the emergency department when a 65-year-old elderly patient was brought to the emergency room by 115 with respiratory and cardiac arrest. The patient was quickly transferred to the CPR room and resuscitation began, and after half an hour of effort, the patient died and we disconnected him from the devices. All the emergency nurses and medical and nursing students had gathered to see how the patient was resuscitated and they were discussing the resuscitation procedures and sometimes laughing among themselves, meanwhile no one had anything to do with the deceased patient, the students also saw He is not accompanied. They came one by one and practiced intubation on him. What is the duty of the responsible nurse in dealing with this action and why?

4. The patient had come to the hospital with a decrease in hemoglobin and was ordered a pixel unit. The patient was hospitalized in the emergency room. I was working with one of my colleagues in the observation room, the nurse mistakenly connected the pixel room to the patient on the side bed. Before the nurse left the room, the patient receiving blood became short of breath, the nurse immediately realized that she had connected the pixel incorrectly and immediately stopped the infusion and informed the doctor. The doctor prescribed hydrocortisol for the patient and the patient was not told anything about this error. Is it the right thing to hide this issue from the patient and why?

5. While caring for the patient's pain relief, you realize that the patient is addicted to drugs, and in this conversation, the patient asks you not to tell anyone about his secret? What do you do in this situation? Why?

6. Consider a situation where you and one of your colleagues are taking care of patients in your department. A 70-year-old patient in a bad mood, whose family's economic and social level is not high, dies due to the wrong injection of medicine by your colleague, no one except you and your colleague knows about this matter. You know that if this mistake had not been made, this patient would have survived despite being sick. What do you do in this situation? Why?
