# Supplementary material for: The effect and comparison of training in ethical decision-making through lectures and group discussions on moral reasoning, moral distress and moral sensitivity in nurses: a clinical randomized controlled trial
Source: BMC Med Ethics. 2023 Aug 4;24:58. doi: 10.1186/s12910-023-00938-5 (PMC10403849; doi:10.1186/s12910-023-00938-5)
Supplement: Supplementary file 2 — Additional File 2: Table s1–Table s11. [file 12910_2023_938_MOESM2_ESM.docx]

**Table S1:** Distribution the total scores of nursing dilemma, moral distress and moral sensitivity according to sociodemographic characteristics of participants (n=245)

| Variables | | **N (%)** | **NPT score** | **P-value** | **Familiarity score** | **P-value** | **MD score** | **P-value** | **MS score** | **P-value** |
| --- | --- | --- | --- | --- | --- | --- | --- | --- | --- | --- |
| Gender | Male | 78 (31.8) | 40.23±7.77 | 0.369 | 14.05±3.99 | 0.192 | 61.10±24.18 | 0.856 | 62.08±11.95 | 0.083 |
|  | Female | 167 (68.2) | 41.06±6.16 |  | 13.32±4.13 |  | 60.45±27.21 |  | 64.57±9.64 |  |
| Age (year) | ≤ 40 | 143 (58.4) | 40.89±6.89 | 0.800 | 13.65±4.05 | 0.654 | 61.62±26.68 | 0.500 | 62.94±9.91 | 0.142 |
|  | > 40 | 102 (41.6) | 40.67±6.49 |  | 13.41±4.16 |  | 59.31±25.66 |  | 64.94±11.16 |  |
| Marital status | Single | 32 (13.1) | 43.13±7.60 | **0.035*** | 14.22±4.25 | 0.323 | 58.31±28.89 | 0.589 | 62.59±11.41 | 0.495 |
|  | Married | 213 (86.9) | 40.45±6.52 |  | 13.45±4.06 |  | 61.01±25.87 |  | 63.95±10.34 |  |
| Work experience | ≤ 15 | 127 (51.8) | 41.58±7.17 | 0.057 | 13.69±4.04 | 0.596 | 62.30±26.79 | 0.310 | 62.73±10.07 | 0.106 |
| (year) | > 15 | 118 (48.2) | 39.90±6.10 |  | 13.41±4.15 |  | 58.89±25.62 |  | 64.90±10.82 |  |
| Ward of | General | 132 (53.9) | 41.56±6.47 | 0.054 | 13.49±3.72 | 0.809 | 63.23±26.35 | 0.098 | 64.24±10.51 | 0.452 |
| working | ICU | 113 (46.1) | 39.90±6.91 |  | 13.62±4.50 |  | 57.65±25.89 |  | 63.23±10.45 |  |
| Position | Head nurse | 34 (13.9) | 41.00±7.66 | 0.793 | 12.94±4.01 | 0.271 | 63.41±22.35 | 0.801 | 64.65±11.08 | 0.142 |
|  | In charge nurse | 65 (26.5) | 40.31±5.51 |  | 13.09±4.24 |  | 60.51±27.38 |  | 63.75±10.89 |  |
|  | Nurse | 146 (59.6) | 40.97±6.99 |  | 13.90±4.03 |  | 60.08±26.67 |  | 63.58±10.20 |  |
| Employment | Full time | 140 (57.1) | 40.35±6.56 | 0.452 | 13.60±3.96 | 0.773 | 62.63±26.54 | 0.396 | 62.82±10.36 | 0.082 |
| types | Part time | 12 (4.9) | 40.67±8.44 |  | 14.25±4.55 |  | 57.08±21.33 |  | 60.75±13.69 |  |
|  | Contract employees | 93 (38) | 41.48±6.71 |  | 13.39±4.26 |  | 58.15±26.32 |  | 65.60±10.02 |  |
| Shift working | Fixed shift | 148 (60.4) | 40.77±6.53 | 0.941 | 13.29±3.98 | 0.219 | 61.49±25.24 | 0.542 | 64.51±9.54 | 0.178 |
|  | Rotation shift | 97 (39.6) | 40.84±7.02 |  | 13.95±4.24 |  | 59.39±27.77 |  | 62.66±11.72 |  |
| Overtime work | ≤ 60 | 133 (54.3) | 41.35±6.55 | 0.157 | 13.54±4.12 | 0.968 | 60.86±26.36 | 0.897 | 64.31±9.75 | 0.387 |
| (hours) | > 60 | 112 (45.7) | 40.13±6.87 |  | 13.56±4.07 |  | 60.42±26.19 |  | 63.14±11.28 |  |
| Awareness of | Completely | 47 (19.2) | 41.81±7.14 | 0.153 | 13.09±3.98 | 0.536 | 61.02±27.11 | 0.993 | 64.17±10.87 | 0.772 |
| Ethical code | Partly | 126 (51.4) | 41.12±6.80 |  | 13.82±4.17 |  | 60.65±24.73 |  | 63.31±10.01 |  |
|  | Never | 72 (29.4) | 39.57±6.17 |  | 13.39±4.04 |  | 60.43±28.48 |  | 64.33±11.010 |  |
| Awareness of | Completely | 183 (74.7) | 40.52±6.53 | 0.556 | 13.33±4.09 | 0.236 | 61.47±26.80 | 0.204 | 63.97±10.45 | 0.522 |
| Patients' Rights | Partly | 58 (23.7) | 41.59±6.92 |  | 14.33±4.12 |  | 59.64±24.55 |  | 63.55±10.55 |  |
|  | Never | 4 (1.6) | 41.75±12.20 |  | 12.50±2.88 |  | 58.25±14.56 |  | 58.00±11.22 |  |
| History of attending to ethics courses | No | 153 (62.4) | 40.63±6.62 | 0.627 | 13.61±3.89 | 0.780 | 61.68±26.17 | 0.433 | 63.78±10.48 | 0.987 |
|  | Yes | 92 (37.6) | 41.07±6.89 |  | 13.46±4.41 |  | 58.95±26.39 |  | 63.75±10.52 |  |
| Total of participants | | 245 (100) | 40.80±6.71 | - | 13.55±4.09 | - | 60.66±26.23 | - | 63.78±10.47 | - |

*P<0.05 considered as significant; NPT: Nursing Principled Thinking; MD: Moral Distress; MS: Moral Sensitivity; P-values were represented by independent-sample t-test and one-way ANOVA for two and three categorized variables, respectively.

**Table S2:** Distribution the scores of moral distress's dimensions according to sociodemographic characteristics of participants (n=245)

| Variables | | **N (%)** | **Inappropriate competencies and responsibilities** | **P-value** | **Errors** | **P-value** | **Not respecting the ethics principles** | **P-value** |
| --- | --- | --- | --- | --- | --- | --- | --- | --- |
| Gender | Male | 78 (31.8) | 18.78±8.27 | 0.575 | 23.26±10.07 | 0.887 | 19.06±8.54 | 0.824 |
|  | Female | 167 (68.2) | 18.05±9.95 |  | 23.05±10.51 |  | 19.34±9.30 |  |
| Age (year) | ≤ 40 | 143 (58.4) | 18.41±9.25 | 0.814 | 23.54±11.06 | 0.453 | 19.67±9.04 | 0.393 |
|  | > 40 | 102 (41.6) | 18.12±9.73 |  | 22.53±9.308 |  | 18.67±9.06 |  |
| Marital status | Single | 32 (13.1) | 17.59±10.77 | 0.657 | 22.44±10.48 | 0.691 | 18.28±10.47 | 0.516 |
|  | Married | 213 (86.9) | 18.39±9.24 |  | 23.22±10.36 |  | 19.40±8.38 |  |
| Work experience | ≤ 15 | 127 (51.8) | 19.09±9.33 | 0.169 | 23.57±10.73 | 0.475 | 19.64±9.14 | 0.491 |
| (year) | > 15 | 118 (48.2) | 17.42±9.52 |  | 22.63±9.96 |  | 18.84±8.97 |  |
| Ward of | General | 132 (53.9) | 19.28±9.48 | 0.075 | 24.14±10.21 | 0.096 | 19.81±8.78 | 0.298 |
| working | ICU | 113 (46.1) | 17.12±9.29 |  | 21.93±10.44 |  | 18.60±9.35 |  |
| Position | Head nurse | 34 (13.9) | 19.94±9.16 | 0.527 | 22.97±8.96 | 0.977 | 20.50±7.83 | 0.643 |
|  | In charge nurse | 65 (26.5) | 17.75±9.77 |  | 23.35±10.44 |  | 19.40±9.22 |  |
|  | nurse | 146 (59.6) | 18.14±9.37 |  | 23.05±10.68 |  | 18.90±9.26 |  |
| Employment | Full time | 140 (57.1) | 19.54±9.46 | 0.054 | 23.67±10.31 | 0.588 | 19.41±9.13 | 0.949 |
| types | Part time | 12 (4.9) | 16.83±8.53 |  | 21.33±9.56 |  | 18.92±7.09 |  |
|  | Contract employees | 93 (38) | 16.58±9.31 |  | 22.52±10.56 |  | 19.05±9.22 |  |
| Shift working | Fixed shift | 148 (60.4) | 18.92±9.12 | 0.195 | 23.23±9.99 | 0.836 | 19.34±8.80 | 0.857 |
|  | Rotation shift | 97 (39.6) | 17.32±9.87 |  | 22.95±10.94 |  | 19.12±9.45 |  |
| Overtime work | ≤ 60 | 133 (54.3) | 18.42±9.50 | 0.807 | 22.88±10.54 | 0.696 | 19.56±9.10 | 0.569 |
| (hours) | > 60 | 112 (45.7) | 18.13±9.40 |  | 23.40±10.17 |  | 18.89±9.01 |  |
| Awareness of | Completely | 47 (19.2) | 18.09±9.80 | 0.977 | 23.91±9.96 | 0.717 | 19.02±9.25 | 0.617 |
| Ethical code | Partly | 126 (51.4) | 18.26±8.94 |  | 22.61±10.12 |  | 19.78±8.58 |  |
|  | Never | 72 (29.4) | 18.46±10.14 |  | 23.49±11.08 |  | 18.49±9.76 |  |
| Awareness of | Completely | 183 (74.7) | 18.45±9.69 | 0.322 | 23.51±10.51 | 0.067 | 19.51±9.15 | 0.589 |
| Patients' Rights | Partly | 58 (23.7) | 18.26±8.66 |  | 22.96±9.74 |  | 18.69±8.85 |  |
|  | Never | 4 (1.6) | 11.25±7.54 |  | 11.50±4.04 |  | 15.50±8.06 |  |
| History of attending to ethics courses | No | 153 (62.4) | 18.46±9.511 | 0.714 | 23.54±10.53 | 0.417 | 19.69±8.93 | 0.335 |
|  | Yes | 92 (37.6) | 18.00±9.366 |  | 22.42±10.07 |  | 18.53±9.24 |  |
| Total participants | | 245 (100) | 18.29±9.44 | - | 23.12±10.35 | - | 19.25±9.05 | - |

P-values were represented by independent-sample t-test and one-way ANOVA for two and three categorized variables, respectively

**Table S3:** Distribution the scores of moral sensitivity's dimensions according to sociodemographic characteristics of participants (n=245)

| Variables | **Respect the patient’s**  **autonomy** | **P-**  **value** | **Communicate with the patient** | **P-value** | **Professional knowledge** | **P-value** | **Ethical problems and conflicts** | **P-value** | **Meaning** | **P-value** | **Benevolence** | **P-value** |
| --- | --- | --- | --- | --- | --- | --- | --- | --- | --- | --- | --- | --- |
| Male | 8.96±2.29 | **0.001*** | 14.64±3.41 | **0.001*** | 3.67±1.91 | 0.914 | 7.99±2.20 | **0.007*** | 12.15±3.17 | 0.061 | 15.50±4.06 | 0.375 |
| Female | 10.08±1.733 |  | 16.45±3.80 |  | 3.64±1.65 |  | 8.71±1.81 |  | 11.37±2.95 |  | 15.95±3.53 |  |
| Age ≤ 40 | 9.69±1.92 | 0.751 | 15.58±3.30 | 0.139 | 3.52±1.70 | 0.184 | 8.36±1.94 | 0.239 | 11.66±3.06 | 0.790 | 15.45±3.60 | 0.071 |
| Age > 40 | 9.77±2.09 |  | 16.30±4.32 |  | 3.82±1.77 |  | 8.66±1.98 |  | 11.56±3.03 |  | 16.31±3.80 |  |
| Single | 10.22±1.35 | 0.135 | 16.50±2.68 | 0.321 | 3.06±1.62 | **0.040*** | 8.41±1.89 | 0.817 | 10.72±3.72 | 0.072 | 15.38±4.36 | 0.479 |
| Married | 9.65±2.04 |  | 15.79±3.90 |  | 3.74±1.73 |  | 8.49±1.98 |  | 11.76±2.91 |  | 15.87±3.60 |  |
| Experience ≤ 15 | 9.60±1.95 | 0.298 | 15.37±3.31 | **0.027*** | 3.62±1.73 | 0.802 | 8.33±1.97 | 0.213 | 11.62±3.01 | 0.993 | 15.45±3.72 | 0.115 |
| Experience > 15 | 9.86±2.03 |  | 16.43±4.14 |  | 3.68±1.74 |  | 8.64±1.95 |  | 11.62±3.08 |  | 16.19±3.66 |  |
| General ward | 9.85±2.01 | 0.302 | 16.23±4.02 | 0.113 | 3.07±1.67 | **0.002*** | 8.49±2.03 | 0.926 | 11.55±3.03 | 0.709 | 15.77±3.70 | 0.845 |
| ICU ward | 9.58±1.97 |  | 15.47±3.42 |  | 3.97±1.74 |  | 8.47±1.89 |  | 11.70±3.06 |  | 15.86±3.72 |  |
| Head nurse | 9.68±2.11 | 0.799 | 15.91±3.08 | 0.893 | 3.94±1.45 | 0.026* | 8.44±2.25 | 0.977 | 11.71±3.43 | 0.511 | 16.65±3.24 | 0.365 |
| In charge nurse | 9.88±1.73 |  | 16.06±3.47 |  | 4.05±1.54 |  | 8.52±1.99 |  | 11.25±3.19 |  | 15.66±3.98 |  |
| nurse | 9.67±2.08 |  | 15.79±4.04 |  | 3.40±1.84 |  | 8.47±1.89 |  | 11.77±2.87 |  | 15.68±3.69 |  |
| Full time employee | 9.48±2.09 | **0.001*** | 15.36±3.37 | **0.002*** | 3.76±1.67 | 0.301 | 8.29±1.99 | 0.129 | 11.64±3.07 | 0.435 | 15.61±3.68 | 0.571 |
| Part time employee | 8.17±2.91 |  | 14.17±3.43 |  | 3.00±1.95 |  | 8.25±2.98 |  | 12.67±2.96 |  | 15.67±4.27 |  |
| Contract employee | 10.30±1.47 |  | 16.89±4.15 |  | 3.57±1.79 |  | 8.81±1.73 |  | 11.46±3.01 |  | 16.13±3.67 |  |
| Fixed shift | 9.73±1.90 | 0.975 | 15.82±2.98 | 0.769 | 3.76±1.66 | 0.231 | 8.57±1.99 | 0.399 | 12.01±2.82 | **0.012*** | 15.90±3.61 | 0.638 |
| Rotation shift | 9.72±2.13 |  | 15.97±4.73 |  | 3.48±1.83 |  | 8.35±1.92 |  | 11.02±3.26 |  | 15.67±3.85 |  |
| ≤ 60 overtime | 10.03±1.85 | **0.009*** | 16.34±4.03 | **0.038*** | 3.71±1.75 | 0.571 | 8.48±1.95 | 0.997 | 11.44±2.71 | 0.302 | 15.77±3.51 | 0.850 |
| > 60 overtime | 9.37±2.10 |  | 15.34±3.36 |  | 3.58±1.72 |  | 8.48±1.99 |  | 11.84±3.39 |  | 15.86±3.93 |  |
| Awareness of ethical code | |  |  |  |  |  |  |  |  |  |  |  |
| Completely | 9.68±1.88 | 0.096 | 15.64±3.15 | 0.882 | 4.06±1.83 | 0.178 | 8.53±2.10 | 0.558 | 11.85±3.33 | 0.424 | 15.96±3.53 | 0.862 |
| Partly | 9.97±1.72 |  | 15.96±3.15 |  | 3.53±1.75 |  | 8.36±2.03 |  | 11.37±3.02 |  | 15.68±3.74 |  |
| Never | 9.33±2.42 |  | 15.90±4.98 |  | 3.58±1.61 |  | 8.67±1.74 |  | 11.90±2.88 |  | 15.93±3.78 |  |
| Awareness of Patients' Rights | |  |  |  |  |  |  |  |  |  |  |  |
| Completely | 9.88±1.92 | 0.067 | 16.15±3.89 | 0.153 | 3.73±1.71 | 0.170 | 8.52±1.98 | 0.786 | 11.44±3.01 | 0.291 | 15.82±3.68 | 0.908 |
| Partly | 9.34±2.17 |  | 15.07±3.11 |  | 3.48±1.79 |  | 8.38±1.98 |  | 12.16±2.96 |  | 15.83±3.83 |  |
| Never | 8.25±1.50 |  | 15.25±5.67 |  | 2.25±1.25 |  | 8.00±0.01 |  | 12.00±4.96 |  | 15.00±3.74 |  |
| History of attending to ethics courses | |  |  |  |  |  |  |  |  |  |  |  |
| No | 9.67±1.94 | 0.590 | 15.57±3.99 | 0.466 | 3.55±1.71 | 0.246 | 8.44±1.95 | 0.703 | 11.78±3.13 | 0.297 | 15.91±3.69 | 0.586 |
| Yes | 9.82±2.08 |  | 16.11±3.36 |  | 3.82±1.77 |  | 8.54±1.99 |  | 11.36±2.88 |  | 15.64±3.74 |  |
| Total participants | 9.73±1.99 | - | 15.88±3.76 | - | 3.65±1.73 | - | 8.48±1.96 | - | 11.62±3.04 | - | 15.81±3.70 | - |

**Table S4:** Nurse's responses to section A of the Nursing Dilemma Test (n=245)

| Scenarios | **‘What should nurse do?’** | **n (%)** |
| --- | --- | --- |
| 1. Newborn with anomalies | Should resuscitate the newborn | 160 (65.3) |
|  | Cannot decide | 42 (17.1) |
|  | Should not resuscitate the newborn | 43 (17.6) |
| 2. Forcing medication | Should forcefully give the medication | 61 (24.9) |
|  | Cannot decide | 10 (4.1) |
|  | Should not forcefully give the medication | 174 (71) |
| 3. Adults’ requests to die | Should aid with artificial respiration | 229 (93.5) |
|  | Cannot decide | 6 (2.4) |
|  | Should not aid with artificial respiration | 10 (4.1) |
| 4. New nurse orientation | Should allocate time for orientation of the nurse | 83 (33.9) |
|  | Cannot decide | 21 (8.6) |
|  | Should not allocate time for orientation of the nurse | 141 (57.6) |
| 5. Medication error | Should report the medication error now | 222 (90.6) |
|  | Cannot decide | 17 (6.9) |
|  | Should not report the medication error now | 6 (2.4) |
| 6. Terminally ill adults | Should answer the patient’s questions | 109 (44.5) |
|  | Cannot decide | 38 (15.5) |
|  | Should not answer the patient’s questions | 98 (40) |

**Table S5:** Univariate and multivariate binary logistic regression analysis to show the association of sociodemographic characteristic with the section C (familiarity score ≤18 vs. >18) of the Nursing Dilemma Test

| Sociodemographic characteristics | P-value | OR | 95% CI | |
| --- | --- | --- | --- | --- |
|  |  |  | **Lower** | **Upper** |
| **Univariate analysis** |  |  |  |  |
| Age | 0.701 | 1.008 | 0.967 | 1.052 |
| Age group (>40 years vs. <40 years) | 0.516 | 1.23 | 0.659 | 2.297 |
| Gender (male vs. female) | 0.171 | 1.554 | 0.827 | 2.923 |
| Marital status (single vs. married) | 0.62 | 1.244 | 0.524 | 2.956 |
| Work experience (>15 years vs. <15 years) | 0.433 | 1.277 | 0.692 | 2.357 |
| Wards (ICU vs. general) | 0.863 | 1.055 | 0.574 | 1.941 |
| Position (head nurse vs. nurse) | 0.113 | 2.455 | 0.81 | 7.441 |
| Position (head nurse vs. in charge nurse) | 0.46 | 1.309 | 0.64 | 2.676 |
| Employee (full time vs. contract) | 0.766 | 1.05 | 0.762 | 1.445 |
| Shift (fixed vs. rotation) | 0.204 | 0.672 | 0.364 | 1.241 |
| Overtime (<60 h vs. >60h) | 0.374 | 0.998 | 0.994 | 1.002 |
| Awareness of Ethic code (completely vs. never) | 0.898 | 0.956 | 0.478 | 1.912 |
| Awareness of Ethic code (completely vs. partly) | 0.56 | 0.876 | 0.562 | 1.366 |
| Awareness of Patients' Rights (completely vs. never) | 0.577 | 0.84 | 0.454 | 1.553 |
| Attending to ethics course (yes vs. no) | 0.322 | 0.732 | 0.394 | 1.358 |
| **Multivariate analysis** |  |  |  |  |
| Age group (<40 years vs. >40 years) | 0.808 | 0.89 | 0.347 | 2.283 |
| Gender (male vs. female) | 0.201 | 1.664 | 0.763 | 3.629 |
| Marital status (single vs. married) | 0.443 | 1.433 | 0.571 | 3.598 |
| Work experience (>15 years vs. <15 years) | 0.755 | 1.157 | 0.464 | 2.884 |
| Wards (ICU vs. general) | 0.849 | 1.032 | 0.745 | 1.43 |
| Position (head nurse vs. nurse) | 0.125 | 2.563 | 0.77 | 8.533 |
| Position (head nurse vs. in charge nurse) | 0.391 | 1.405 | 0.646 | 3.06 |
| Employee (full time vs. part time) | 0.98 | 1.005 | 0.688 | 1.467 |
| Employee (full time vs. contract) | 0.963 | 1.036 | 0.234 | 4.592 |
| Shift (fixed vs. rotation) | 0.246 | 0.683 | 0.359 | 1.3 |
| Awareness of Ethic code (completely vs. never) | 0.481 | 0.833 | 0.502 | 1.383 |
| Awareness of Patients' Rights (completely vs. never) | 0.865 | 0.941 | 0.468 | 1.893 |
| Attending to ethics course (yes vs. no) | 0.195 | 0.634 | 0.318 | 1.264 |

OR: Odds Ratio, CI: Confidence intervals

**Table S6:** Univariate and multivariate binary logistic regression analysis to show the association of sociodemographic characteristic with score of the moral distress scale (≤58 vs. >58)

| Sociodemographic characteristics | P-value | OR | 95% CI | |
| --- | --- | --- | --- | --- |
|  |  |  | **Lower** | **Upper** |
| Univariate analysis |  |  |  |  |
| Age group (<40 years vs. >40 years) | 0.359 | 1.27 | 0.763 | 2.113 |
| Gender (male vs. female) | 0.76 | 1.087 | 0.635 | 1.862 |
| Marital status (single vs. married) | 0.337 | 0.691 | 0.325 | 1.469 |
| Work experience (>15 years vs. <15 years) | 0.27 | 0.754 | 0.456 | 1.246 |
| Wards (ICU vs. general) | 0.319 | 0.88 | 0.684 | 1.132 |
| Position (head nurse vs. nurse) | 0.6 | 1.221 | 0.578 | 2.58 |
| Position (head nurse vs. in charge nurse) | 0.973 | 0.99 | 0.551 | 1.777 |
| Employee (full time vs. part time) | 0.229 | 1.382 | 0.816 | 2.34 |
| Employee (full time vs. contract) | 0.874 | 1.006 | 0.868 | 3.064 |
| Shift (fixed vs. rotation) | 0.817 | 1.062 | 0.637 | 1.773 |
| Overtime (<60 h vs. >60h) | 0.505 | 1.187 | 0.717 | 1.963 |
| Awareness of Ethic code (completely vs. never) | 0.577 | 1.233 | 0.591 | 2.575 |
| Awareness of Ethic code (completely vs. partly) | 0.648 | 1.145 | 0.641 | 2.046 |
| Awareness of Patients' Rights (completely vs. never) | 0.109 | 1.644 | 0.377 | 1.102 |
| Attending to ethics course (yes vs. no) | 0.479 | 0.829 | 0.494 | 1.392 |
| Multivariate analysis |  |  |  |  |
| Age group (<40 years vs. >40 years) | 0.968 | 1.016 | 0.478 | 2.158 |
| Gender (male vs. female) | 0.537 | 1.23 | 0.637 | 2.375 |
| Marital status (single vs. married) | 0.348 | 0.68 | 0.304 | 1.521 |
| Work experience (>15 years vs. <15 years) | 0.156 | 0.565 | 0.257 | 1.243 |
| Wards (ICU vs. general) | 0.349 | 0.878 | 0.67 | 1.152 |
| Position (head nurse vs. nurse) | 0.714 | 0.928 | 0.622 | 1.385 |
| Position (head nurse vs. in charge nurse) | 0.838 | 0.935 | 0.491 | 1.781 |
| Employee (full time vs. part time) | 0.086 | 1.744 | 0.924 | 3.291 |
| Employee (full time vs. contract) | 0.789 | 1.193 | 0.328 | 4.333 |
| Shift (fixed vs. rotation) | 0.83 | 1.061 | 0.618 | 1.821 |
| Overtime (<60 h vs. >60h) | 0.774 | 1.099 | 0.578 | 2.088 |
| Awareness of Ethic code (completely vs. never) | 0.522 | 1.316 | 0.568 | 3.054 |
| Awareness of Ethic code (completely vs. partly) | 0.601 | 1.179 | 0.636 | 2.186 |
| Awareness of Patients' Rights (completely vs. never) | 0.084 | 1.595 | 0.33 | 1.073 |
| Attending to ethics course (yes vs. no) | 0.24 | 0.705 | 0.393 | 1.264 |

OR: Odds Ratio, CI: Confidence intervals

**Table S7:** Univariate and multivariate binary logistic regression analysis to show the association of sociodemographic characteristic with scores of the moral sensitivity (≤75 vs. >75)

| Sociodemographic characteristics | P-value | OR | 95% CI | |
| --- | --- | --- | --- | --- |
|  |  |  | **Lower** | **Upper** |
| Univariate analysis |  |  |  |  |
| Age | 0.066 | 1.068 | 0.996 | 1.147 |
| Age group (<40 years vs. >40 years) | 0.089 | 0.444 | 0.175 | 1.13 |
| Gender (male vs. female) | 0.752 | 0.856 | 0.328 | 2.238 |
| Marital status (single vs. married) | 0.789 | 0.838 | 0.231 | 3.039 |
| Work experience (>15 years vs. <15 years) | 0.273 | 1.684 | 0.663 | 4.277 |
| Wards (ICU vs. general) | 0.302 | 0.777 | 0.482 | 1.254 |
| Position (head nurse vs. nurse) | 0.137 | 2.136 | 0.785 | 5.815 |
| Position (head nurse vs. in charge nurse) | 0.578 | 1.473 | 0.377 | 5.76 |
| Employee (contract vs. full time) | 0.242 | 1.324 | 0.827 | 2.12 |
| Shift (fixed vs. rotation) | 0.969 | 1.019 | 0.4 | 2.591 |
| Overtime (<60 h vs. >60h) | 0.593 | 1.289 | 0.508 | 3.275 |
| Awareness of Ethic code (completely vs. never) | 0.392 | 0.63 | 0.218 | 1.815 |
| Awareness of Ethic code (completely vs. partly) | 0.871 | 1.105 | 0.329 | 3.712 |
| Awareness of Patients' Rights (completely vs. never) | 0.867 | 1.099 | 0.364 | 3.314 |
| Attending to ethics course (yes vs. no) | 0.469 | 1.444 | 0.535 | 3.899 |
| Multivariate analysis |  |  |  |  |
| Age | 0.685 | 1.028 | 0.901 | 1.172 |
| Age group (<40 years vs. >40 years) | 0.416 | 0.466 | 0.074 | 2.929 |
| Gender (male vs. female) | 0.307 | 0.547 | 0.172 | 1.74 |
| Marital status (single vs. married) | 0.76 | 0.803 | 0.196 | 3.286 |
| Work experience (>15 years vs. <15 years) | 0.889 | 1.113 | 0.248 | 4.99 |
| Wards (ICU vs. general) | 0.251 | 0.74 | 0.443 | 1.237 |
| Position (head nurse vs. in charge nurse) | 0.796 | 1.225 | 0.263 | 5.703 |
| Position (head nurse vs. nurse) | 0.254 | 1.898 | 0.631 | 5.708 |
| Employee (full time vs. contract) | 0.101 | 0.373 | 0.115 | 1.21 |
| Employee (full time vs. part time) | 0.527 | 0.467 | 0.044 | 4.962 |
| Shift (fixed vs. rotation) | 0.952 | 1.032 | 0.368 | 2.889 |
| Awareness of Ethic code (completely vs. partly) | 0.792 | 1.207 | 0.299 | 4.872 |
| Awareness of Ethic code (completely vs. never) | 0.941 | 0.972 | 0.463 | 2.043 |
| Awareness of Ethic code (completely vs. partly) | 0.918 | 0.992 | 0.463 | 2.001 |
| Awareness of Patients' Rights (completely vs. never) | 0.867 | 1.099 | 0.364 | 3.314 |
| Attending to ethics course (yes vs. no) | 0.251 | 1.021 | 1.171 | 2.586 |

OR: Odds Ratio, CI: Confidence intervals

**Table S8:** Unadjusted and adjusted binary logistic regression analysis to evaluate association between three groups of study with the section B (NPT score ≤50 vs. >50) of the Nursing Dilemma Test

| **Variables** | **P-value** | **OR** | **95% CI** | |
| --- | --- | --- | --- | --- |
|  |  |  | **Lower** | **Upper** |
| **Unadjusted** |  |  |  |  |
| Groups (discussion group vs. lecture group) | **<0.001*** | **3.029** | **1.005** | **5.171** |
| Groups (lecture group vs. control group) | **<0.001*** | **9.329** | **2.005** | **14.171** |
| Groups (discussion group vs. control group) | **<0.001*** | **11.083** | **6.484** | **15.461** |
| Age group (>40 years vs. <40 years) | 0.101 | 2.857 | 0.815 | 10.015 |
| Gender (female vs. male) | 0.286 | 2.109 | 0.535 | 8.321 |
| Marital status (married vs. single) | 0.383 | 0.542 | 0.137 | 2.147 |
| Work Experience (>15 years vs. 15 years) | 0.571 | 1.414 | 0.427 | 4.685 |
| Wards groups (ICU vs. general) | 0.242 | 0.485 | 0.144 | 1.630 |
| Position (head nurse vs. in charge nurse) | 0.702 | 1.364 | 0.278 | 6.683 |
| Position (head nurse vs. nurse) | 0.900 | 0.909 | 0.206 | 4.015 |
| Employee types (full time vs. part time) | 0.228 | 1.457 | 0.128 | 2.632 |
| Employee types (full time vs. contract) | 0.290 | 0.267 | 0.023 | 3.080 |
| Shift groups (rotation vs. fixed) | 0.759 | 0.821 | 0.231 | 2.910 |
| Overtime (>60 hours vs. <60 hours) | 0.132 | 0.389 | 0.114 | 1.328 |
| Code of Ethics (completely vs. never) | 0.268 | 4.200 | 0.332 | 5.123 |
| Code of Ethics (completely vs. partly) | 0.586 | 1.941 | 0.178 | 2.119 |
| Patient rights (completely vs. partly) | 0.711 | 0.796 | 0.239 | 2.659 |
| Patient rights (completely vs. partly/never) | 0.989 | 1.006 | 0.239 | 2.659 |
| **Adjusted** |  |  |  |  |
| Groups (discussion group vs. lecture group) | **0.008*** | **13.078** | **3.238** | **15.954** |
| Groups (lecture group vs. control group) | **<0.001*** | **14.329** | **2.005** | **16.171** |
| Groups (discussion group vs. control group) | **<0.001*** | **18.01** | **5.834** | **22.154** |
| Age group (>40 years vs. <40 years) | 0.574 | 2.937 | 0.069 | 5.822 |
| Gender (female vs. male) | 0.332 | 8.68 | 0.111 | 18.751 |
| Marital status (married vs. single) | 0.423 | 0.281 | 0.013 | 6.273 |
| Work experience (>15 years vs. 15 years) | 0.245 | 9.623 | 0.212 | 11.673 |
| Wards groups (ICU vs. general) | 0.138 | 0.034 | 0.001 | 2.954 |
| Position (head nurse vs. in charge nurse) | 0.859 | 1.187 | 0.180 | 7.804 |
| Employee types (full time vs. part time) | 0.302 | 2.937 | 0.379 | 4.756 |
| Shift groups (rotation vs. fixed) | 0.745 | 0.651 | 0.049 | 8.593 |
| Overtime (>60 hours vs. <60 hours) | 0.997 | 0.992 | 0.025 | 3.688 |
| Code of ethics (completely vs. partly/never) | 0.985 | 1.027 | 0.071 | 4.782 |
| Patient rights (completely vs. partly/never) | 0.985 | 1.027 | 0.071 | 4.782 |

*P<0.05 considered as significant, OR: Odds Ratio, CI: Confidence intervals

**Table S9:** Unadjusted and adjusted binary logistic regression analysis to evaluate association between three groups of study with the section C (familiarity score ≤18 vs. >18) of the Nursing Dilemma Test

| **Variables** | **P-value** | **OR** | **95% CI** | |
| --- | --- | --- | --- | --- |
|  |  |  | **Lower** | **Upper** |
| **Unadjusted** |  |  |  |  |
| Groups (discussion group vs. Lecture group) | 0.728 | 1.275 | 0.324 | 5.013 |
| Groups (Lecture group vs. Control group) | 0.474 | 0.593 | 0.141 | 2.484 |
| Groups (discussion group vs. Control group) | 0.709 | 0.756 | 0.173 | 3.294 |
| Age group (>40 years vs. <40 years) | 0.217 | 0.417 | 0.104 | 1.673 |
| Gender (female vs. male) | 0.348 | 0.444 | 0.082 | 2.42 |
| Marital status (married vs. single) | 0.841 | 1.172 | 0.249 | 5.507 |
| Work experience (>15 years vs. 15 years) | 0.161 | 0.917 | 0.813 | 1.035 |
| Wards groups (ICU vs. general) | 0.387 | 0.538 | 0.132 | 2.193 |
| Position (head nurse vs. in charge nurse) | 0.253 | 3.706 | 0.392 | 5.009 |
| Position (head nurse vs. nurse) | 0.168 | 4.765 | 0.518 | 5.798 |
| Employee types (full time vs. part time) | 0.398 | 0.385 | 0.042 | 3.523 |
| Employee types (full time vs. contract) | 0.472 | 1.731 | 0.388 | 7.725 |
| Shift groups (rotation vs. fixed) | 0.362 | 0.522 | 0.129 | 2.116 |
| Overtime (>60 hours vs. <60 hours) | 0.301 | 2.100 | 0.514 | 8.573 |
| Code of ethics (completely vs. partly /never) | **0.029*** | **0.173** | **0.036** | **0.840** |
| Patient rights (completely vs. partly or never) | 0.073 | 0.302 | 0.082 | 1.118 |
| **Adjusted** |  |  |  |  |
| Groups (discussion group vs. Lecture group) | 0.481 | 0.371 | 0.024 | 5.840 |
| Groups (lecture group vs. control group) | 0.304 | 0.342 | 0.044 | 2.641 |
| Groups (discussion group vs. control group) | 0.876 | 0.855 | 0.121 | 6.059 |
| Age group (>40 years vs. <40 years) | **0.035*** | **0.073** | **0.006** | **0.833** |
| Gender (female vs. male) | 0.501 | 0.285 | 0.007 | 1.046 |
| Marital status (married vs. single) | 0.494 | 0.369 | 0.021 | 6.448 |
| Work experience (>15 years vs. 15 years) | **0.040*** | **0.021** | **0.001** | **0.841** |
| Wards groups (ICU vs. general) | 0.656 | 1.735 | 0.154 | 9.583 |
| Position (head nurse vs. in charge nurse) | 0.190 | 0.356 | 0.076 | 1.668 |
| Employee types (full time vs. part time) | 0.269 | 0.400 | 0.079 | 2.030 |
| Shift groups (rotation vs. fixed) | 0.228 | 0.237 | 0.023 | 2.460 |
| Overtime (>60 hours vs. <60 hours) | 0.890 | 0.84 | 0.072 | 9.841 |
| Code of ethics (completely vs. partly/never) | 0.070 | 0.067 | 0.004 | 1.252 |
| Patient rights (completely vs. partly/never) | 0.175 | 0.189 | 0.017 | 2.095 |

*P<0.05 considered as significant, OR: Odds Ratio, CI: Confidence intervals

**Table S10:** Unadjusted and adjusted binary logistic regression analysis to evaluate association between three groups of study with the with score of the moral distress scale (≤58 vs. >58)

| **Variables** | **P-value** | **OR** | **95% CI** | |
| --- | --- | --- | --- | --- |
|  |  |  | **Lower** | **Upper** |
| **Unadjusted** |  |  |  |  |
| Groups (discussion group vs. Lecture group) | 0.500 | 0.630 | 0.165 | 2.41 |
| Groups (Lecture group vs. Control group) | 0.132 | 0.389 | 0.114 | 1.328 |
| Groups (discussion group vs. Control group) | **0.034*** | **0.245** | **0.067** | **0.902** |
| Age group (>40 years vs. <40 years) | 0.347 | 1.909 | 0.497 | 7.337 |
| Gender (female vs. male) | 0.088 | 6.600 | 0.755 | 7.724 |
| Marital status (married vs. single) | 0.438 | 0.560 | 0.130 | 2.421 |
| Work experience (>15 years vs. 15 years) | 0.391 | 1.800 | 0.470 | 6.898 |
| Wards groups (ICU vs. general) | **0.009*** | **0.105** | **0.019** | **0.565** |
| Position (head nurse vs. in charge nurse) | 0.668 | 1.429 | 0.279 | 7.302 |
| Position (head nurse vs. nurse) | 0.423 | 2.000 | 0.366 | 3.919 |
| Employee types (full time vs. Contract) | 0.714 | 1.312 | 0.307 | 5.620 |
| Employee types (part time vs. contract) | 0.276 | 3.500 | 0.368 | 5.308 |
| Shift groups (rotation vs. fixed) | 0.948 | 0.955 | 0.234 | 3.888 |
| Overtime (>60 hours vs. <60 hours) | 0.999 | 1.001 | 0.265 | 3.769 |
| Code of ethics (completely vs. partly /never) | **0.030*** | **0.231** | **0.061** | **0.869** |
| Patient rights (completely vs. partly or never) | 0.715 | 0.773 | 0.194 | 3.082 |
| **Adjusted** |  |  |  |  |
| Groups (discussion group vs. Lecture group) | 0.873 | 0.868 | 0.152 | 4.954 |
| Groups (Lecture group vs. Control group) | **0.021*** | **0.105** | **0.015** | **0.717** |
| Groups (discussion group vs. Control group) | **0.009*** | **0.089** | **0.015** | **0.547** |
| Age group (>40 years vs. <40 years) | 0.763 | 1.487 | 0.113 | 9.604 |
| Gender (female vs. male) | 0.085 | 3.242 | 0.610 | 6.595 |
| Marital status (married vs. single) | 0.072 | 0.045 | 0.002 | 1.324 |
| Work experience (>15 years vs. 15 years) | 0.817 | 0.701 | 0.034 | 4.318 |
| Wards groups (ICU vs. general) | **0.010*** | **0.031** | **0.002** | **0.435** |
| Position (head nurse vs. nurse) | 0.958 | 1.004 | 0.248 | 4.363 |
| Employee types (full time vs. part time) | 0.125 | 0.295 | 0.062 | 1.405 |
| Shift groups (rotation vs. fixed) | 0.590 | 2.031 | 0.155 | 6.688 |
| Overtime (>60 hours vs. <60 hours) | 0.088 | 2.483 | 0.690 | 5.906 |
| Code of ethics (completely vs. partly/never) | 0.141 | 0.191 | 0.021 | 1.732 |
| Patient rights (completely vs. partly/never) | 0.099 | 6.745 | 0.698 | 8.152 |

*P<0.05 considered as significant, OR: Odds Ratio, CI: Confidence intervals

**Table S11:** Unadjusted and adjusted binary logistic regression analysis to evaluate association between three groups of study with the with scores of the moral sensitivity (≤75 vs. >75)

| **Variables** | **P-value** | **OR** | **95% CI** | |
| --- | --- | --- | --- | --- |
|  |  |  | **Lower** | **Upper** |
| **Unadjusted** |  |  |  |  |
| Groups (discussion group vs. Lecture group) | **0.007*** | **7.600** | **1.732** | **10.347** |
| Groups (Lecture group vs. Control group) | 0.540 | 1.458 | 0.436 | 4.880 |
| Groups (discussion group vs. Control group) | **0.002*** | **11.083** | **2.484** | **14.461** |
| Age group (>40 years vs. <40 years) | 0.894 | 0.917 | 0.256 | 3.286 |
| Gender (female vs. male) | 0.324 | 0.475 | 0.108 | 2.084 |
| Marital status (married vs. single) | 0.854 | 1.143 | 0.275 | 4.756 |
| Work experience (>15 years vs. 15 years) | 0.919 | 1.067 | 0.306 | 3.718 |
| Wards groups (ICU vs. general) | 0.245 | 2.125 | 0.597 | 7.568 |
| Position (head nurse vs. in charge nurse) | 0.385 | 2.200 | 0.371 | 3.038 |
| Position (head nurse vs. nurse) | 0.224 | 2.933 | 0.518 | 16.61 |
| Employee types (full time vs. part time) | 0.842 | 0.875 | 0.236 | 3.241 |
| Employee types (full time vs. contract) | 0.747 | 1.500 | 0.127 | 7.667 |
| Shift groups (rotation vs. fixed) | 0.939 | 1.053 | 0.282 | 3.935 |
| Overtime (>60 hours vs. <60 hours) | 0.343 | 1.846 | 0.521 | 6.547 |
| Code of ethics (completely vs. partly /never) | 0.484 | 1.483 | 0.493 | 4.461 |
| Patient rights (completely vs. partly or never) | 0.785 | 1.193 | 0.336 | 4.236 |
| **Adjusted** |  |  |  |  |
| Groups (discussion group vs. Lecture group) | **0.005*** | **10.874** | **6.043** | **12.886** |
| Groups (Lecture group vs. Control group) | 0.433 | 1.758 | 0.429 | 7.21 |
| Groups (discussion group vs. Control group) | **0.002*** | **13.077** | **8.454** | **16.774** |
| Age group (>40 years vs. <40 years) | 0.473 | 0.433 | 0.044 | 4.269 |
| Gender (female vs. male) | 0.421 | 0.308 | 0.017 | 5.429 |
| Marital status (married vs. single) | 0.597 | 0.554 | 0.062 | 4.934 |
| Work experience (>15 years vs. 15 years) | 0.663 | 1.782 | 0.133 | 23.877 |
| Wards groups (ICU vs. general) | 0.679 | 0.579 | 0.044 | 7.696 |
| Position (head nurse vs. nurse) | **0.040*** | **1.985** | **1.008** | **4.898** |
| Employee types (full time vs. part time) | 0.742 | 1.278 | 0.298 | 5.486 |
| Shift groups (rotation vs. fixed) | 0.873 | 1.206 | 0.121 | 12.07 |
| Overtime (>60 hours vs. <60 hours) | 0.190 | 5.149 | 0.445 | 59.561 |
| Code of ethics (completely vs. partly/never) | **0.034*** | **4.959** | **1.222** | **8.057** |
| Patient rights (completely vs. partly/never) | 0.320 | 4.556 | 0.229 | 90.586 |

*P<0.05 considered as significant, OR: Odds Ratio, CI: Confidence intervals
